# Targeting neutrophil extracellular traps in cancer progression and metastasis

**DOI:** 10.7150/thno.111096

**Published:** 2025-04-22

**Authors:** Ji Zhang, Changhong Miao, Hao Zhang

**Affiliations:** 1 Department of Anesthesiology, Zhongshan Hospital, Fudan University, Shanghai, China.; 2 Shanghai Key Laboratory of Perioperative Stress and Protection, Shanghai, China.; 3 Department of Anesthesiology, Shanghai Medical College, Fudan University, China.

**Keywords:** neutrophil extracellular traps, cancer progression, metastasis, tumor microenvironment, therapeutic targets

## Abstract

Neutrophils serve as pivotal effectors and regulators of the intricate immune system. Their contributions are indispensable, encompassing the obliteration of pathogens and a significant role in both cancer initiation and progression. Conversely, malignancies profoundly affect neutrophil activity, maturation, and lifespans. Cancer cells manipulate their biology to enhance or suppress the key functions of neutrophils. This manipulation is one of the most remarkable defensive mechanisms used by neutrophils, including the formation of neutrophil extracellular traps (NETs). NETs are filamentous structures comprising DNA, histones, and proteins derived from cytotoxic granules. In this review, we discuss the bidirectional interplay in which cancer elicits NET formation, and NETs concurrently facilitate cancer progression. Here, we discuss how vascular dysfunction and thrombosis induced by neutrophils and NETs contribute to an elevated risk of mortality from cardiovascular complications in patients with cancer. Ultimately, we propose a series of therapeutic strategies that hold promise for effectively targeting NETs in clinical settings.

## 1. Introduction

Neutrophils represent a pivotal category of white blood cells found within the circulatory system, comprising 50%-70% of the total leukocyte population, and serve as frontline defenders of the immune response 1. These formidable cells primarily perform their roles by orchestrating the recruitment of additional immune entities through phagocytosis, inciting the generation of reactive oxygen species (ROS), modulating the degradation and release of proteases or other cytotoxic agents, and activating fellow immune components 2, 3. Moreover, they engage in sophisticated defense mechanisms, known as neutrophil extracellular traps (NETs), among various functions aimed at combating invading pathogens 4. The process by which neutrophils generate NETs is termed NETosis, and can be divided into two categories: suicidal and active. The former denotes a condition in which neutrophils succumb during NET formation, whereas active NETosis allows the retention of viable neutrophilic functionalities 5. As discovered by Brinkmann *et al.*, NETs comprise a novel defensive strategy used by neutrophils; their chemical composition predominantly comprises chromatin, deoxyribonucleic acid (DNA), and protein 6, 7. NETs function not only as instruments through which neutrophils ensnare and eradicate pathogens, but also as pathological indicators associated with various diseases due to their excessive release 8. Notable examples include preeclampsia, chronic inflammatory disorders, and myocardial ischemia-reperfusion injuries, which culminate in the overwhelming discharge of NETs.

**Figure 1:**
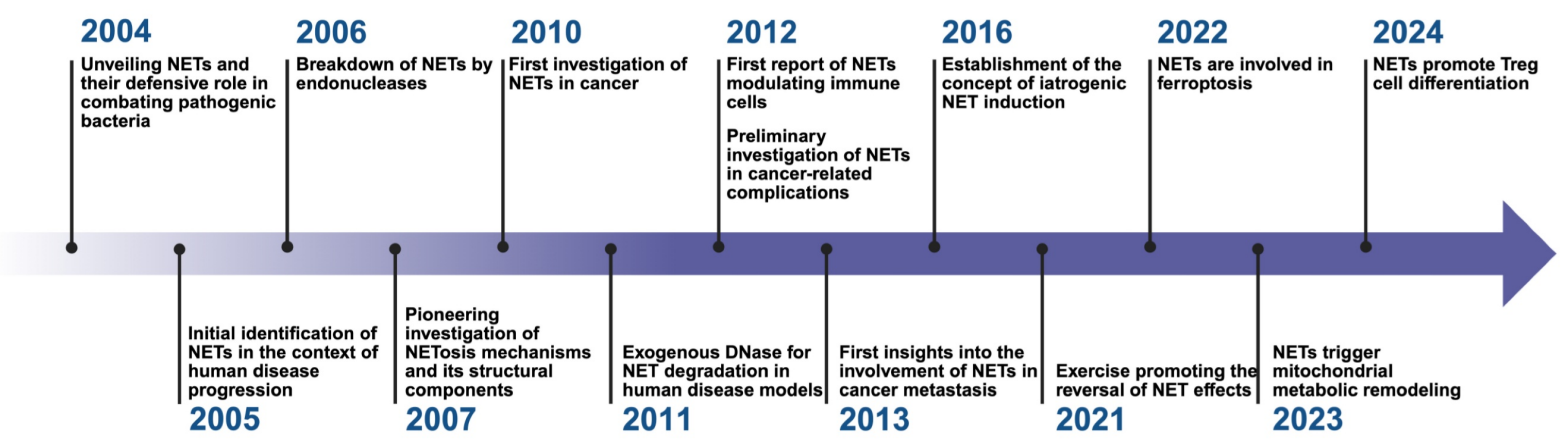
** Research history of NETs.** This timeline highlights significant milestones in the research and understanding of Neutrophil Extracellular Traps (NETs) from 2004 to 2024. The timeline illustrates the evolution of key discoveries and their impact on various domains, particularly in the field of oncology and immunology. *Created with BioRender.com.

An essential biomarker of tumorigenesis is a heightened inflammatory milieu, exemplified by the augmented release of NETs 9. Excessive secretion of NETs results in chronic inflammation throughout the body. An abundance of NETs fosters tumor angiogenesis through mechanisms such as degradation of the extracellular matrix, entrapment of circulating tumor cells, compromise of vascular integrity, and induction of epithelial-mesenchymal transition (EMT), thereby creating an opportune "premetastatic microenvironment" conducive to both tumor initiation and metastatic spread 10. Nonetheless, neoplastic cells adeptly elude their primary site, infiltrate the bloodstream, evade the immune system facilitated by NETs, and subsequently establish colonies at distal sites 11. This surge in NETs production engenders a permissive microenvironment that supports tumor cell proliferation and expedites metastasis 12. Given this understanding, modulating the expression levels of NETs may inhibit tumor cell growth while simultaneously dismantling the metastatic landscape, which has emerged as a novel avenue for cancer therapy 11. Recent investigations have revealed that NETs can potentiate the immunosuppressive cell functions while undermining the functions of CD8+ T and natural killer (NK) cells. Consequently, this dual effect compromises anti-tumor immunity and accentuates oncogenic processes 12. Given the intricate nature of the Tumor Microenvironment (TME), our knowledge of the interplay between NETs and immune responses remains limited, and existing strategies aimed at inhibiting NET formation are usually simple. Such constraints have significantly curtailed the broader application of targeting NETs in contemporary cancer immunotherapy **(Figure 1)**.

Herein, we elucidate the potential triggers and associated signaling cascades that precipitate NET formation, particularly focusing on their implications within the tumor microenvironment. Subsequent evidence has increasingly underscored the critical role of NETs as crucial mediators of various aspects of tumor progression, including awakening of dormant cancer cells, extravasation of circulating tumor cells (CTCs), and recurrence following metastasis. Furthermore, NETs are intricately involved in inflammation-induced disturbances that culminate in the establishment of an immunosuppressive milieu, facilitating immune evasion and promoting tumor survival and proliferation. Considering the multifaceted roles attributed to NETs, it is plausible to consider them not only as prospective prognostic biomarkers but also as promising therapeutic targets in oncology.

## 2. Basic components and functions of NETs

**Figure 2:**
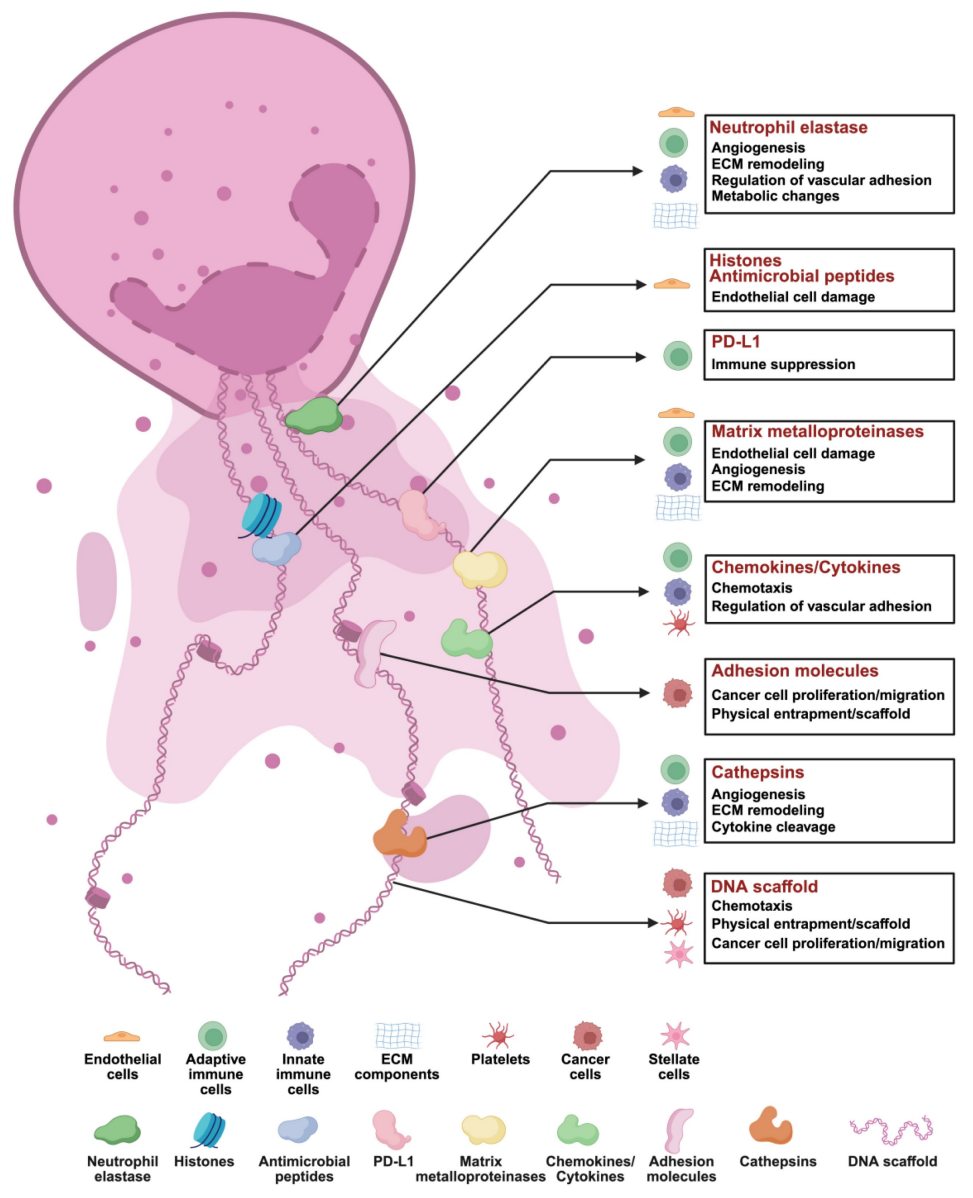
** Composition of NETs.** Neutrophil extracellular traps (NETs) are composed of a DNA scaffold released by neutrophils and a variety of functional proteins that play critical roles in tumor biology, immune modulation, and microenvironment remodeling. The core components and their functions include: (1) Neutrophil Elastase: Facilitates angiogenesis by degrading the extracellular matrix (ECM) and modulates endothelial adhesion, inducing metabolic changes that impact immune cells, ECM, and endothelial cell functions. (2) Histones and Antimicrobial Peptides: Directly cause endothelial cell damage and exhibit antimicrobial activity against pathogens. (3) Programmed Death-Ligand 1 (PD-L1): Suppresses immune responses by modulating adaptive immune cells. (4) Matrix Metalloproteinases (MMPs): Promote endothelial damage, angiogenesis, and ECM remodeling through degradation, influencing immune cells, ECM, and endothelial cell dynamics. (5) Chemokines and Cytokines: Recruit immune cells and platelets by inducing chemotaxis and regulating vascular adhesion, thereby orchestrating inflammation and tumor progression. (6) Adhesion Molecules: Act as scaffolds or physical barriers for immune cells, promoting cancer cell proliferation, migration, and immune evasion. (7) Cathepsins: Contribute to angiogenesis, ECM remodeling, and cytokine cleavage, modulating immune responses and ECM architecture. (8) DNA Scaffold: Provides structural support and physical entrapment for immune and tumor cells, while generating chemotactic signals that influence tumor migration and differentiation. *Created with BioRender.com.

In 2004, Brinkmann *et al.* published a groundbreaking article in *Science* that elucidated the fundamental architecture and function of NETs for the first time. This pioneering study revealed that NETs represent an intricate extracellular network composed of chromatin intertwined with a diverse array of protein constituents, designed to restrain and obliterate invading pathogens 6. The myriad functions of NETs are associated with their constituent components. Over 200 proteins have been identified within these structures 13, underscoring the complexity of their function. For instance, NETs have been implicated in disease severity, such as bronchiectasis severity index, quality of life, future risk of hospital admission, and mortality in patients with bronchiectasis 14. NF-κB-dependent autoimmunity induced by NETs-DNA via cGAS/TLR9 in chronic obstructive pulmonary disease 15. Furthermore, they play a pivotal role in the pathogenesis of rheumatoid arthritis by inducing carbamylation and histone-mediated osteoclast formation 16. Besides chronic inflammation and autoimmune disorders, emerging evidence suggests that NETs contribute to tumorigenesis. Zhan *et al.* found HBV-induced S100A9 activates RAGE/TLR4-ROS signaling, leading to abundant NETs formation, which subsequently promoted the growth and metastasis of hepatocellular carcinoma (HCC) cells 17. In addition, HMGB1 present within NETs has been found to accelerate the progression of diffuse large B-cell lymphoma and colorectal cancer via the TLR9-MAPK signaling pathways 18, 19. Beyond their protein constituents, DNA elements contained within NETs can initiate the β-parvin-RAC1-CDC42 signaling pathway through the membrane receptor Coiled-coil domain containing 25(CCDC25), mediating metastasis in MDA-MB-231 cells 20. Collectively, these investigations highlight that NETs can influence tumor advancement from a multifaceted perspective; however, their complex composition poses significant challenges to related research. Future studies should adopt a holistic approach to examine the roles of various NET components in tumor progression. Moreover, it is essential to further delineate how these structures impact tumor immunity, potentially positioning this line of inquiry among upcoming priorities **(Figure 2)**.

## 3. Mechanism of NETs formation

As early as 1996, Takei *et al.* made significant observations, revealing that phorbol-12-myristate-13-acetate (PMA) could induce distinct morphological alterations in neutrophils that are divergent from the processes of apoptosis and necrosis. This pioneering study serves as a foundation for future studies related to NETs 21. Currently, NET formation involves two principal pathways: NETosis and Non-lytic NETosis. Regarding morphology and functionality, neutrophils undergoing NETosis exhibit marked loss of their characteristic cellular structure and activity upon NET formation. Conversely, cells undergoing Non-lytic NETosis retain their transient phagocytic capabilities following NET production. Regarding the stimuli and mechanisms underpinning these processes, PMA, antibodies, and cholesterol crystallization can facilitate the progression of NETosis by inducing ROS, which instigates chromatin depolymerization. In contrast, Staphylococcus aureus and Escherichia coli promote Non-lytic NETosis through vesicular release of protein-modified chromatin via TLR2 and TLR4 along a non-reactive oxygen species-dependent pathway 22-24. Nevertheless, whether functional disparities exist between differentially formed NETs remains an area that warrants further investigation. Chromatin depolymerization constitutes a critical prerequisite for NET formation, and studies have identified peptidyl arginine deiminase 4 (PAD4) as an integral part of this process. The disruption of NET formation observed in PAD4 knockout mice underscores its indispensable role in facilitating connections within pathways, leading to net assembly 25, 26. PAD4 catalyzes the citrullination of histone H3's arginine residues while simultaneously inhibiting their binding affinity to negatively charged DNA backbones. This promotes chromatin densification followed by depolymerization, which is crucial for effective NET development 27. Moreover, intracellular ROS levels have been shown to influence neurogenesis. NADPH oxidase-derived or mitochondria-associated ROS accelerates chromatin depolymerization by synergistically activating myeloperoxidase (MPO) alongside neutrophil elastase (NE), propelling these factors into the nucleus 28-30.

**Figure 3:**
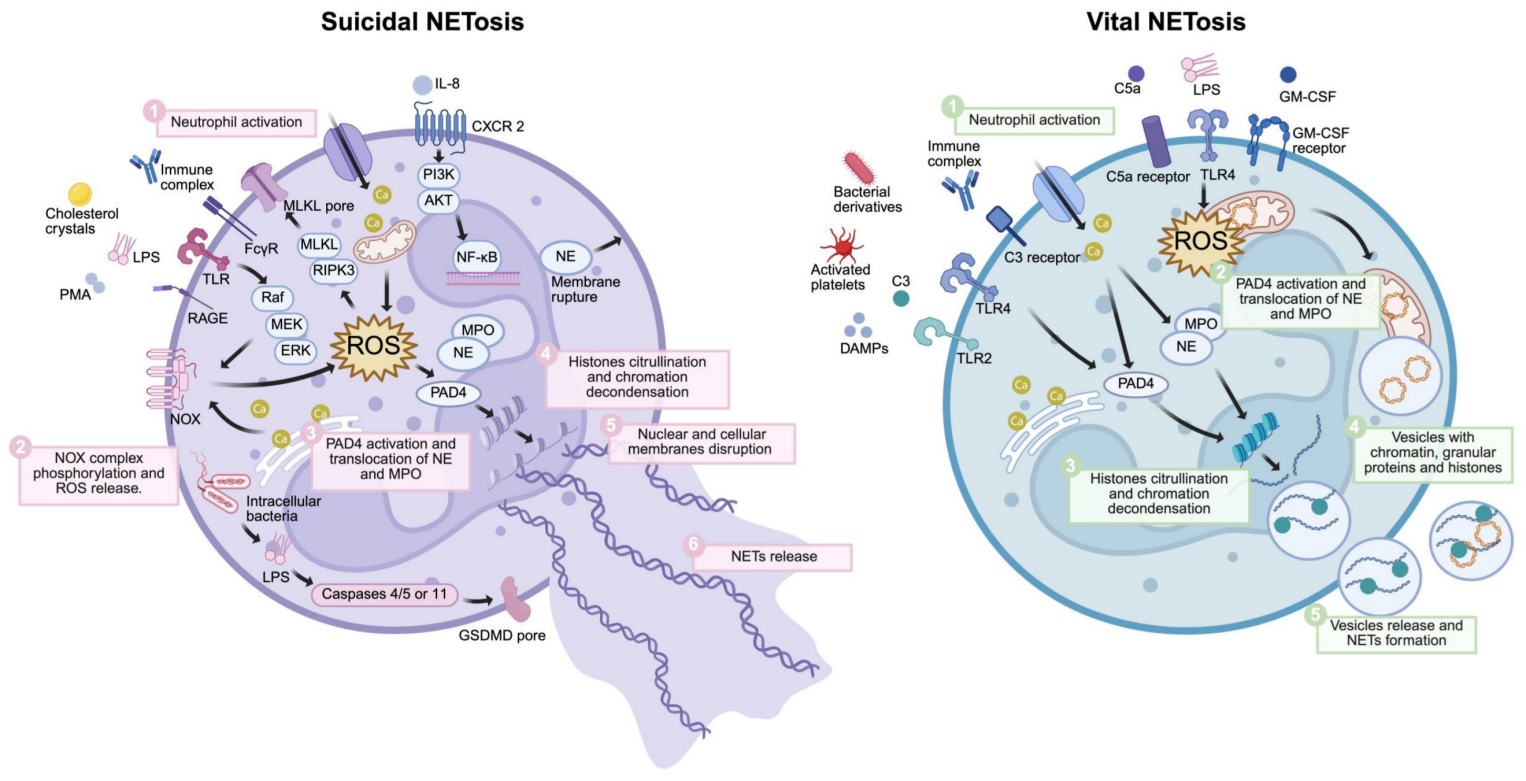
** Formation of NETs.** The formation of NETs includes suicide NETosis and vital NETosis. Suicidal NETosis (left) is initiated by stimuli such as PMA, LPS, immune complexes, cholesterol crystals, or IL-8. These extracellular signals mainly activate the NOX complex via several pathways, such as the Raf-MEK-ERK signaling pathways. IL-8 also activates NF-κB via PI3K-AKT pathways. The influx of extracellular calcium ions can activate mitochondria. These signaling pathways subsequently generate ROS in the cytoplasm, resulting in the release of NE and MPO from azurophilic granules, activation of PAD4, and their translocation into the cell nucleus. Subsequently, activated PAD4 catalyzes the citrullination of histones and chromatin decondensation with the aid of NE and MPO. Additionally, ROS can both activate RIPK3-MLKL, leading to membrane perforation, and cause membrane rupture through NE release. Moreover, intracellular bacterial LPS forms GSDMD pores via caspases 4/5 or 11. When the nuclear membrane breaks down, the decondensed chromatin enters the cytoplasm, mixes with granular proteins, and forms NETs. Finally, NETs are released following the membrane and the mechanical action of swollen chromatin. Vital NET formation (right) is initiated by stimuli such as S. aureus, DAMPs, LPS, activated platelets, and bacterial derivatives. One process is mainly mediated by Ca²⁺ but is independent of the NOX complex. Activated PAD4, NE, and MPO also translocate into the nucleus to promote chromatin decondensation. Mitochondria participate in another pathway by releasing mtDNA and generating mtROS. Lastly, NETs, which may include nuclear DNA and mtDNA, are stored within vesicles budding from nuclei and released by neutrophils without membrane rupture. Abbreviations: LPS, lipopolysaccharide; PMA, phorbol-12-myristate-13-acetate; RAGE, Receptor for Advanced Glycation End-products; FcR, Fc receptor; IL-8, interleukin-8; DAMPs, damage-associated molecular patterns; TLR, toll-like receptor; MPO, myeloperoxidase; NE, neutrophil elastase; NOX, NADPH oxidase; PAD4, protein-arginine deiminase 4; ROS, reactive oxygen species; GSDMD, gasdermin D; mtDNA, mitochondrial DNA; MLKL, mixed lineage kinase domain-like protein.*Created with BioRender.com.

Besides directly facilitating NET formation, neutrophil elastase possesses the capacity to activate Gasdermin D (GSDMD), further enhancing NET production 31. Moreover, DEK functions analogously to MPO, inducing NET formation after binding to DNA 32. Collectively, these studies showed that the intricate process of NETosis in neutrophils is orchestrated by a plethora of crucial proteins, among which PAD4 has emerged as a pivotal target and has been extensively studied for its role in promoting NET formation. Numerous contemporary investigations have corroborated that various PAD4 inhibitors can markedly disrupt NET synthesis *in vivo*, while concurrently impeding tumor progression 33-35. Furthermore, the precise mechanisms that govern neutrophils within the tumor microenvironment remain unclear. Consequently, probing whether functional variances exist between NETs formed via disparate pathways could prove advantageous for targeted inhibition strategies aimed at mitigating the undesired off-target effects associated with inhibitors. In addition, determining whether tumor heterogeneity influences the dynamics of NET formation is a principal focus for guiding future research on NETs **(Figure 3)**.

### 3.1. Suicidal NETosis

Pathogens, including bacteria, fungi, viruses, neutrophil cytoplasmic antibodies, and nutate and bacterial lipopolysaccharides, along with calcium ionophoresis and other stimuli, can induce neutrophils to release NETs 36. The intricate process of NETs release is initiated by the engagement of cellular receptors that prompt the efflux of calcium ions from the endoplasmic reticulum. This cascade subsequently activates protein kinase C and NADPH oxidase complex, helping to generate ROS 27. ROS then stimulates PAD4, which catalyzes the conversion of arginine residues on histones into citrulline. Citrullinated histones facilitate chromatin depolymerization and compaction, orchestrating NET formation 37. Moreover, proteins such as neutrophil granuloprotein and MPO play pivotal roles in directing NE toward nuclear translocation 38. This translocation results in the concomitant release of condensed chromatin and granular materials into the extracellular milieu, ultimately leading to NET extrusion via the disruption of the plasma membrane. This process culminates in neutrophil demise post-NET formation. Within the tumor microenvironment, endothelial cells that produce interleukin-8 (IL-8) and tumor cell-derived granulocyte colony-stimulating factor (G-CSF) mediate NETs formation. Research conducted by Park *et al.* and Gupta *et al.* revealed that co-culturing activated endothelial cells with neutrophils precipitated notable NETs production, a phenomenon partially driven by IL-8 secreted from these stimulated endothelial cells 39, 40. Conversely, G-CSF is often overexpressed within malignant domains, which leads to increased peripheral neutrophilia coupled with enhanced ROS production and subsequent induction of NETosis 41, 42.

### 3.2. Vital NETosis

NET genesis independent of NADPH oxidase occurs because of a lack of ROS production and cell death following stimulation, a phenomenon called non-soluble NETosis 43, 44. In this alternative pathway for NET formation, which bypasses NADPH oxidase activation, neutrophils are stimulated by various factors, including bacteria, bacterial derivatives, activated platelets, and complement proteins. PAD4 plays a pivotal role in promoting chromatin condensation 26, 37. Subsequently, NETs associate with granule proteins and cytoplasmic components before being extruded from the cell via exocytosis while preserving membrane integrity. Remarkably, upon NET release, neutrophils retain their viability and continue to exhibit phagocytic activity and chemotactic responsiveness 45, 46.

### 3.3 Crosstalk between NETs and cancer cells

Recent evidence suggests bidirectional interactions between NETs and cancer cells 39, 47-49. Various cancer-derived factors contribute to NET formation 50, which, in turn, promotes both hypercoagulability and tumor progression 51, 52. Among these factors, cytokines play a crucial role in modulating NET function. For example, chemotherapy-exposed cancer cells secrete IL-1β, which induces NET formation, subsequently driving cancer cells to undergo EMT 53, 54. IL-8 is another key cytokine involved in this process. In diffuse large B-cell lymphoma (DLBCL), IL-8 secreted by tumor cells binds to its receptor, CXCR2, on neutrophils, promoting NET formation. The newly formed NETs activate the TLR9 pathway in DLBCL cells, thereby facilitating tumor progression 18. Cytokine IL-8, derived from CC cells, triggers NETs in an NADPH oxidase-dependent manner, while NET-associated CG promotes cancer metastasis. Clinically, elevated CG protein expression in tumor tissues is closely associated with poor prognosis in HCC patients 55, 56. Additionally, IL-17 57 and TGF-β 11, secreted by cancer cells, have been reported to exert similar effects. In contrast, Chi3l1 facilitates neutrophil recruitment and NET formation, ultimately enhancing anti-tumor immunity 58.

In addition to cytokines, cancer cell metabolites play a pivotal role in NET formation. In gastric cancer, NETs are driven by a hypoxic tumor microenvironment, thereby promoting tumor growth 59. Moreover, lactate, a key product of the Warburg effect, induces NET formation by regulating gene expression, potentially contributing to tumor progression within the TME 60. Notably, lysine (K)-specific demethylase 6A (KDM6A), a frequently mutated tumor suppressor gene in pancreatic ductal adenocarcinoma (PDAC), has been implicated in NET regulation. Yang *et al.* 61 demonstrated that KDM6A loss correlates with increased tumor-associated neutrophils (TANs) and NETs, both of which are known to promote PDAC progression. Additionally, extracellular RNAs from lung cancer cells activate epithelial cells and may indirectly induce NET formation, contributing to lung cancer oncogenesis 62. Overall, the complex interplay and feedback loops between NETs and cancer cells present numerous potential therapeutic targets, warranting further investigation.

## 4. NETs and primary cancer progression

**Figure 4:**
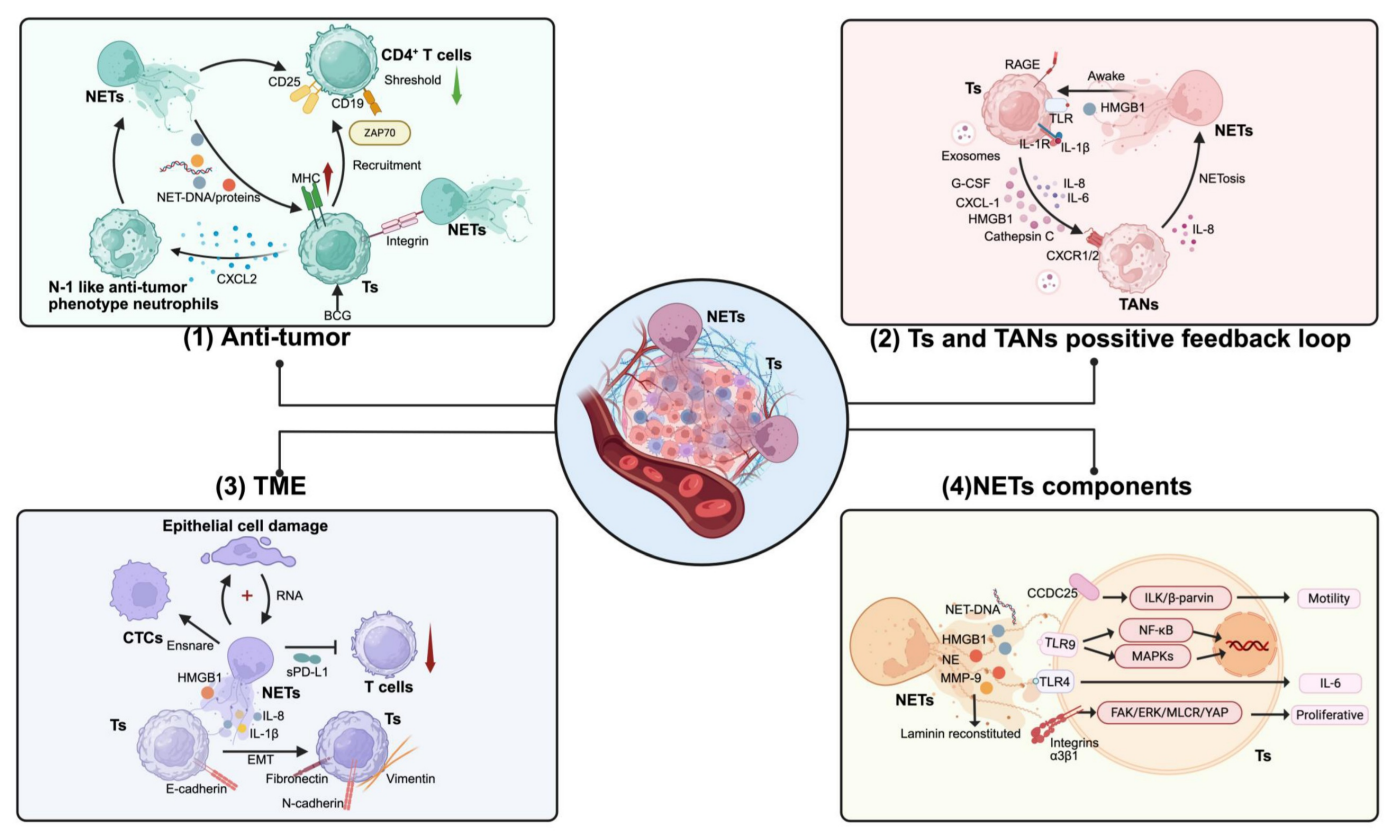
** Role of NETs in the initial progression of cancer.** The impact of Neutrophil Extracellular Traps (NETs) on the initial progression of cancer can be divided into four categories: (1) Anti-tumor Effect: NETs release DNA and associated proteins that are processed and presented by MHC molecules on tumor cells. Tumor cells recruit CD4+ T cells and downregulate their activation threshold through ZAP70. NETs interact with tumor cells and T cells through integrin-mediated adhesion, promoting proximity for effective immune cell-tumor cell interactions. Bacillus Calmette-Guérin (BCG), as an immunotherapeutic agent, augments this response by enhancing the anti-tumor properties of neutrophils. (2) Tumors and Tumor-Associated Neutrophils (TANs) Positive Feedback Loop: Tumors stimulate TANs to secrete IL-8 through exosomes and cellular molecules (G-CSF, CXCL-1, HMGB1, and Cathepsin C), which in turn leads to the formation of NETs. NETs activate tumors via substances such as HMGB1, promoting their differentiation and metastasis. This positive feedback loop forms a malignant cycle, facilitated by molecules such as HMGB1 that activate TANs via TLR and RAGE signaling pathways, inducing further NETosis and secretion of proinflammatory cytokines (IL-1β, IL-6, IL-8), enhancing the recruitment and activation of additional TANs. (3) NETs' Impact on the Tumor Microenvironment: NETs damage epithelial cells and capture circulating tumor cells (CTCs), providing potential support for tumor colonization. They promote tumor migration through the secretion of HMGB1 and cytokines via epithelial-mesenchymal transition (EMT). Additionally, NETs inhibit the cytotoxic activity of T cells through soluble PD-L1 (sPD-L1). (4) Components and Signaling Pathways of NETs in Tumor Cells: NET-derived components, including NET-DNA, HMGB1, neutrophil elastase (NE), and matrix metalloproteinase-9 (MMP-9), interact with tumor cells (Ts) through multiple pathways. CCDC25 and TLR9 recognize NET-DNA, activating the ILK/β-parvin pathway to promote tumor cell motility. TLR4 engagement triggers NF-κB and MAPK signaling cascades. Additionally, reconstituted laminin binds to integrins α3β1, activating the FAK/ERK/MLCR/YAP signaling axis to enhance tumor cell proliferation and IL-6 production. Abbreviations: NETs, Neutrophil Extracellular Traps; Ts, Tumor cells; BCG, Bacillus Calmette-Guérin; MHC, Major Histocompatibility Complex; TANs, Tumor-Associated Neutrophils; G-CSF, Granulocyte Colony-Stimulating Factor; CXCL-1, C-X-C Motif Chemokine Ligand 1; HMGB1, High Mobility Group Box 1; RAGE, Receptor for Advanced Glycation End-products; IL-8, Interleukin 8; TLR, Toll-Like Receptor; CTCs, Circulating Tumor Cells; NE, Neutrophil Elastase; MMP-9, Matrix Metallopeptidase 9; CCDC25, Coiled-Coil Domain Containing 25. *Created with BioRender.com.

Extensive research has demonstrated a strong correlation between NETs and chronic inflammation in the human body, elucidating their capacity to facilitate tumorigenesis by inducing persistent tissue damage and eliciting DNA disruption 63. Following tumor initiation, NETs not only catalyze the proliferation of neoplastic cells through the activation of pivotal signaling pathways 18, providing crucial dynamic support for tumor expansion, but also invigorate mitochondrial biogenesis in cancerous cells and amplify ATP production, providing essential energy reserves for tumor growth 64. Concurrently, NETs may engage in tumor progression via an array of pro-tumor mechanisms. They have been implicated in the induction of EMT 65, endowing malignant cells with invasive and migratory capabilities. NETs facilitate the dissemination of malignancies 66. Their ability to compromise vascular integrity expedites access of neoplastic cells into the circulatory system 67. In addition, NETs enhance the metastatic potential of CTCs by ensnaring them and mediating immune evasion mechanisms 68, 69. Furthermore, studies have shown that NET formation is intricately linked to recurrence in various forms of cancers 70. Specifically, NET-associated NE and Matrix Metalloproteinase 9 (MMP9) possess the capacity to cleave laminin proteins; this action can reawaken dormant breast cancer cell populations from both human origins and murine models, fostering lung metastasis through activation of the Integrinα3β1-FAK-ERK-MLC2 signaling cascade 71. This finding not only uncovered a novel mechanistic pathway whereby NETs contribute to oncological relapse but also provides innovative perspectives toward devising targeted therapeutic strategies aimed at quelling dormant malignant populations. In summary, we assert that NETs play an instrumental role in all phases, encompassing the emergence, evolution, and recurrence of tumors. Targeted interventions against these traps, including strategies geared toward inhibiting their formation, dismantling established NETs structures, or obstructing their pro-oncogenic functionalities, could represent a pioneering approach for curtailing tumor proliferation and thwarting recurrence **(Figure 4)**.

### 4.1. Role of NETs in cancer progression

#### 4.1.1. Anti-tumor effects

The role of NETs in tumor progression is highly context-dependent, shaped by tumor type, microenvironmental factors, and immune interactions. Their anti-tumor effects primarily arise from direct cytotoxicity against malignant cells and immune activation 72. In a comprehensive melanoma biopsy analysis, Schedel *et al.* detected NETs in all 27 ulcerative melanomas but none in seven non-ulcerative cases, with no correlation to disease stage. *In vitro* studies demonstrate that NETs inhibit melanoma cell migration via integrin-mediated adhesion and induce necrosis, though their abundance does not correlate with tumor progression 73. In a pancreatic cancer model, Chan *et al.* 74 found that melatonin induces tumor cells to secrete CXCL2, recruiting TANs, which adopt an N1-like anti-tumor phenotype, release NETs, and promote tumor apoptosis via ROS-dependent NETosis. This suggests that the anti-tumor function of NETs is influenced by neutrophil subtypes and NET formation mechanisms. In addition to neutrophils, immune interactions are critical. CD16⁺ neutrophils promote colorectal cancer by suppressing NK cell activity through NET-mediated NKp46 cleavage 75. Similarly, pancreatic cancer-induced MMP-9 and IDO contribute to NK cell dysfunction 76, highlighting the immunomodulatory role of NET components. Therapeutic interventions targeting NETs have shown promise. An injectable hemostatic gel formulated with a tumor acidity neutralizer and NET lyase has been shown to enhance adoptive NK cell therapy, effectively mitigating post-resection recurrence of hepatocellular carcinoma 77. Interestingly, the role of genes in influencing the effects of NETs on tumor progression is noteworthy. Shen *et al.* 78 utilized gene set enrichment analysis of NET-related gene signatures to evaluate NET levels across various cancer types. Their analysis revealed two distinct survival patterns: in cancers such as prostate, esophageal, breast, and colon cancer, higher NET scores were associated with better survival outcomes, whereas in cancers like pancreatic cancer, lung squamous cell carcinoma, low-grade glioma, ovarian cancer, gastric cancer, and bladder cancer, higher NET scores correlated with poorer survival.

Overall, the effects of NETs on tumor progression are complex and multifactorial. While their role remains debated, increasing evidence suggests they facilitate malignancy under certain conditions. Further elucidation of NET-driven molecular mechanisms may inform novel therapeutic strategies in cancer immunotherapy.

#### 4.1.2. Pro-tumor effects

In the tumor microenvironment, neutrophils are readily activated by various stimuli to generate NETs, which in turn promote tumor proliferation, invasion, and metastasis, creating a deleterious vicious cycle 39, 79. Clinically, elevated plasma levels of NETs have been observed in patients with a range of malignancies, including lung, pancreatic, and bladder cancers, suggesting a direct role in oncogenesis 80. In colorectal cancer, NET presence is independent of tumor location or stage, yet their excessive accumulation is associated with poor patient outcomes 62. NETs also facilitate the reactivation of dormant tumor cells, thus promoting subsequent metastasis 81, as evidenced in models of chronic pulmonary inflammation 82. NETs serve as potent attractants for additional neutrophils that accumulate at the tumor locus while simultaneously ensnaring CTCs. This adhesion allows these cells to attach to vascular walls and subsequently undergo exosmosis. Furthermore, NETs interact with adhesion molecules, such as fibronectin, through proteolytic activity and enlist platelets that envelop CTCs, enhancing immune evasion mechanisms and fostering metastatic spread to distant sites (e.g., the lungs). Ultimately, copious amounts of NETs robustly adhere to blood vessels, providing scaffolding conducive to platelet attachment, activation, and thrombin generation, a process that precipitates thrombosis.

In addition to promoting tumor progression, NETs are implicated in therapeutic resistance and immune suppression. Chemotherapy-induced IL-1β secretion stimulates NET formation, thereby activating TGF-β pathways that mediate chemoresistance 53. Concurrently, NETs upregulate programmed death-ligand 1 (PD-L1), contributing to T cell dysfunction and the establishment of an immunosuppressive tumor microenvironment 83. In ischemia-reperfusion models, blockade of PD-L1 led to significant tumor regression and T cell functional recovery, underscoring the immunosuppressive role of NETs.

### 4.2. NETs components affecting cancer

NETs comprise a diverse array of constituents, including MMP-9, NE, cathepsin G (CG), chemokines, antimicrobial peptides, and histone antibodies. These components are intricately linked to the pathophysiology of endothelial cell injury, angiogenesis, and modulation of vascular adhesion and extracellular matrix (ECM) 84. The factors implicated in cancer cell proliferation and tumor progression include NE, MMP-9, CG, histones, and DNA. In this section, we discuss pivotal elements of NETs and explore their roles in advancing cancer progression.

#### 4.2.1. NE

NE is a serine protease secreted by neutrophils that plays a pivotal role in enhancing phagocytosis and exhibits bactericidal properties, thus serving as an acute-phase protein that is integral to the body's inflammatory response. Remarkably, Cui *et al.* discovered that NE can liberate proteolytic fragments encapsulating death domains by cleaving recombinant CD95 proteins, which subsequently exhibit selective cytotoxicity toward cancer cells upon interaction with the high-abundance histone H1 subtypes present within these malignant cells, underscoring NE's inhibitory influence on tumor cell proliferation 84. Conversely, other studies have revealed that NE may ostensibly facilitate tumor cell proliferation. In studies involving murine models of lung cancer conducted by Houghton *et al.*, it was observed that NE could directly penetrate the cellular endosomal spaces, diminishing insulin receptor substrate expression and encouraging the expansion of lung carcinoma cells 85. Research has linked elevated NE levels with breast cancer metastasis. Consequently, monitoring NE levels may provide prognostic insights in patients with this malignancy 86. In summary, NE has a dual effect on the metastatic processes associated with cancer. It fosters metastasis through intricate remodeling of the TME by stimulating tumor cell growth directly 87, cleaving membrane ligands 88, fostering angiogenesis 88, and synergizing with other components released by the neutrophils 89. Given the complex nature of TME dynamics that influence NE functionality, further research is warranted to ascertain its viability as a clinical prognostic marker 90.

#### 4.2.2. MMP-9

MMP plays pivotal roles in an array of physiological processes, including tissue development, wound healing, tissue remodeling, organ morphogenesis, and angiogenesis. Its expression remains relatively low *in vivo*, and it is subject to stringent regulatory mechanisms. Extensive research has shown that MMP-9 is markedly upregulated in various human malignancies and plays a crucial role in the proliferation and metastasis of cancer cells 91. MMP-9 facilitates the degradation of non-ECM molecules, such as IL-1β and TNF-α. Several studies have substantiated MMP-9's involvement in catalyzing both the onset and metastasis across a spectrum of cancers, including gastric cancer 92, breast cancer 93, 94, colon cancer 95, lung cancer 96, 97, among others.

#### 4.2.3. CG

CG can be synthesized within cells and possesses a remarkable ability to degrade a multitude of intracellular matrix precursor proteins, including collagen, elastin, and laminin. It plays a pivotal role in modulating the secretion and deposition of matrix precursors under normal physiological conditions and in the context of malignancies 98. The hydrolytic activity of CG is significantly influenced by neutrophil-derived DNA, which facilitates its hydrolysis by obstructing the protective effects exerted by endogenous protease inhibitors present in the tissues. Where CG operates in a non-hydrolytic fashion, it exhibits the capacity to activate platelets 99, which subsequently envelops tumor cells and transfers their major histocompatibility complex (MHC) into the same neoplastic cells, resulting in elevated levels of MHC on tumor cell surfaces. This process further activates NK cells, thwarting potential immune evasion strategies used by tumor cells 100 while simultaneously exerting inhibitory effects on them. Additional research suggests that CG may function as a tumor suppressor gene in various malignancies, including breast cancer 101, bladder cancer 102, and colorectal cancer 103. Given that arginine residues embedded within CG sequences dictate whether they act in a hydrolyzed or non-hydrolyzed manner, their propensity to either foster or impede malignant metastasis is contingent on their mode of action. Therefore, the effects of CG on cancer metastasis warrant further investigation.

#### 4.2.4. Histones and DNA

Because histones within NETs possess inherent cytotoxic properties that can directly inflict damage to endothelial cells, they have been shown to elicit proinflammatory signals at certain toxic concentrations, which exceed those induced by DNA alone 104, 105. Furthermore, NET-derived DNA, as a chemokine, induces cancer cell metastasis. In their investigation using a murine sepsis model, Najmh *et al.* 106 discovered that NET-DNA can capture cancerous cells through integrins, facilitating their navigation across the vascular barrier and enabling the colonization of secondary tissues such as the lungs and liver, which helps to promote metastatic spread. In addition, the precursor protein CCDC25 functions as a signal transducer; upon recognizing NET-DNA, it activates the ILK-β-parvin pathway, which subsequently fosters oncogenic metastasis 20. However, the precise mechanisms underpinning how CCDC25 regulates tumor cell behavior remain enigmatic. Tang *et al.* 107 demonstrated that cholesterol enhances CCDC25 expression and revealed an intriguing correlation in which both CCDC25 and fovein-1 were observed to increase and colocalize within 4T1 cells subjected to ASPP2 knockdown. Their findings also indicate that cholesterol inhibitors concurrently diminished the expression of both CCDC25 and fovein-1. Currently, literature pertaining to histones and DNA associated with NETs remains sparse. Existing studies have implied that interactions between histones and DNA may facilitate cancer metastasis. However, thorough elucidation of these specific mechanisms warrants further exploration in future studies.

## 5. Role of NETs in cancer metastasis

**Figure 5:**
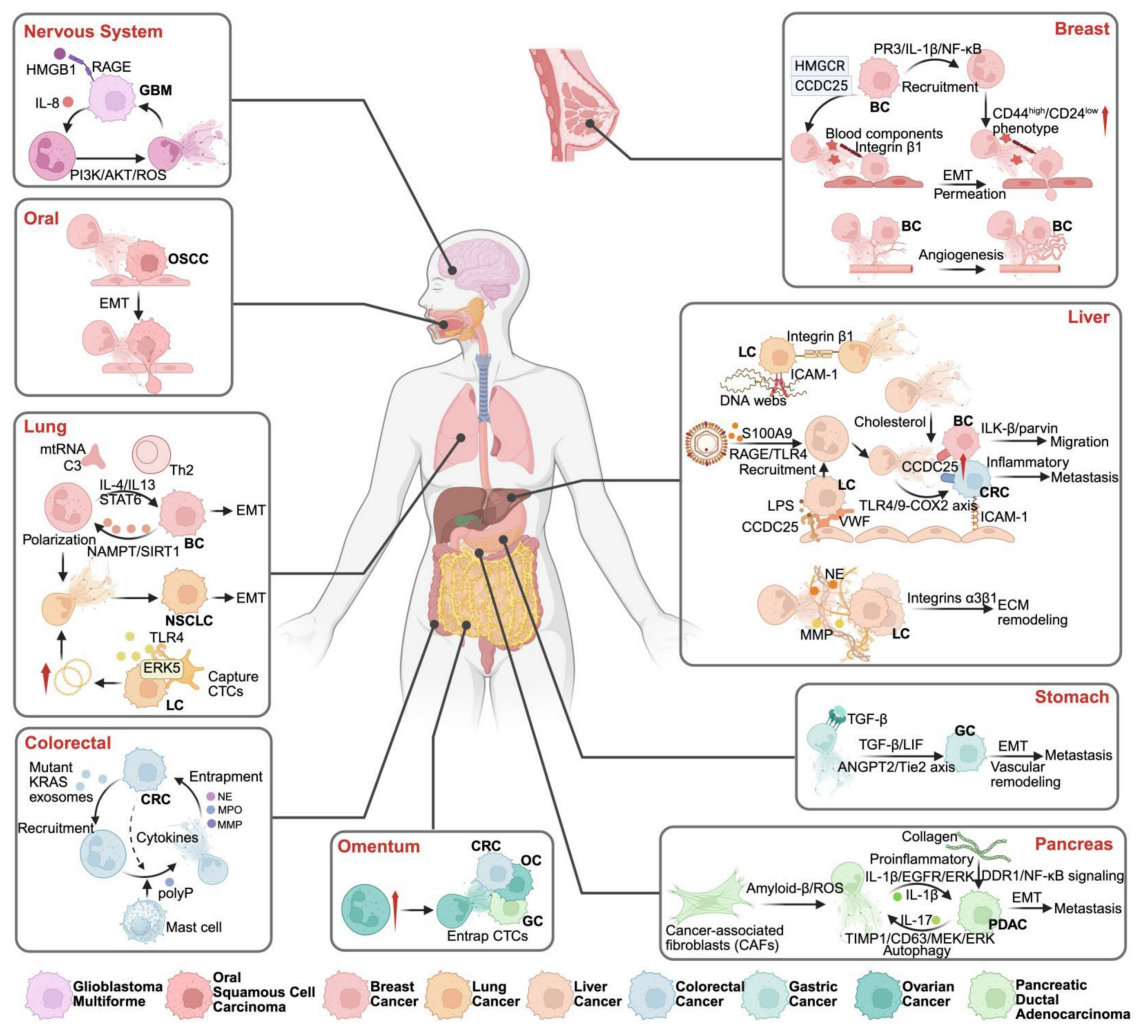
** NET-mediated metastasis in various tumor types.** NETs facilitate tumor metastasis through distinct organ-specific pathways: In the nervous system, GBM metastasis is promoted via HMGB1/RAGE and IL-8/PI3K/AKT/ROS signaling. In OSCC, NETs facilitate cancer progression through the induction of epithelial-mesenchymal transition (EMT), enhancing tumor cell motility and invasiveness. NETs contribute to lung metastasis by supporting polarization of neutrophils and EMT, influenced by cytokines such as IL-4 and IL-13, and pathways including STAT3 and NAMPT/SIRT1. Additionally, lung cancer cells can promote NET formation through the release of mitochondrial DNA. Colorectal cancer metastasis features mutant KRAS-driven NET formation and mast cell interactions. Omental metastasis occurs through NET-mediated CTC entrapment. Breast cancer metastasis involves NET-induced P53/IL-1β/NF-κB signaling, promoting CD44high/CD24low phenotype transition, EMT, and angiogenesis. Liver metastasis is facilitated through multiple NET-dependent pathways: DNA webs activating RAGE/TLR9, cholesterol-mediated mechanisms, and integrin α3β1-dependent ECM remodeling. Gastric cancer metastasis is driven by TGF-β/ANGPT2/Tie2 axis activation. In pancreatic cancer, NETs promote metastasis through inflammatory pathways (IL-1β/IL-17) and collagen-mediated EMT. *Created with BioRender.com.

Single-cell technology has a remarkable capacity to reveal the profound heterogeneity of neutrophils at the cellular level, which may correlate with variations in their migratory capabilities and NET formation at distinct stages throughout their life cycle, as highlighted in several studies 108. Research indicates that NETosis can assume both pro-tumorigenic and anti-tumorigenic roles contingent on TME 109. Given that NETs predominantly facilitate metastasis in various types of cancer, this section elucidates the contribution of these structures to cancer metastasis. Furthermore, NETs play a pivotal role in facilitating the distal metastasis of tumors 110. Although surgical resection remains a fundamental approach to tumor treatment, adverse prognosis is often exacerbated by metastatic progression **(Figure 5)**.

### 5.1. NETs and inflammation

In an *in vivo* model in which mouse LEWIS lung cancer cells were administered intravenously following cecal puncture and ligation, instigating a state of systemic inflammation, it was observed that the entrapment of NETs within the hepatic sinuses, encompassing cancer cells, was significantly correlated with metastatic progression. The introduction of DNases to disintegrate NETs abolished this correlation, supporting the hypothesis that inflammation may be intricately entwined with distant metastasis induced by NETs 111. Previous research has similarly articulated that systemic inflammation augments the adherence of CTCs in peripheral circulation to the hepatic sinuses, consequently facilitating the distant micrometastatic spread of malignancies 112. The integrin β2 present on neutrophils engages intercellular adhesion molecule-1 (ICAM-1) expressed by CTCs, orchestrating neutrophilic modulation that fosters CTC adhesion and obstructs hepatic sinuses, a pivotal element in the metastatic cascade of cancerous cells 113. A study conducted by Yang *et al.* 12 elucidated how an elevated frequency of NETs in patients with hepatocellular carcinoma further propels tumor cell metastasis via the blockade of TLR4/9. In contrast, impairment due to prior inflammatory responses ameliorates NET-mediated metastatic potential. In conclusion, the mechanism by which NETs facilitate tumor cell metastasis appears to be closely associated with inflammatory responses. Nuclear factor-kappa B(NF-κB), an essential transcription factor governing IL-8 expression, is a proinflammatory chemokine that elevates IL-8 secretion within the tumor microenvironment. Zha *et al.* 114 further elucidated that IL-8 expression was elevated in patients with high-grade gliomas. Moreover, HMGB1, a pivotal constituent of NETs, engages with the receptor of advanced glycation end-products to activate the nuclear factor kappa-light-chain-enhancer of NF-κB. This interaction substantiates the notion that NETs facilitate the proliferation, migration, and invasion of tumor cells. Tumor cells reciprocally induce the secretion of IL-8, triggering inflammation and augmenting the infiltration of neutrophils into the tumor tissue, helping to form additional NETs. Although surgical resection remains the most prevalent strategy for oncological intervention, it often carries inherent risks associated with postoperative infections.

In addition, surgical procedures may induce stress responses that alter the procoagulant and inflammatory profiles, which promote tumor metastasis. They documented an alarming increase in NET formation post-surgery in a cohort of patients undergoing hepatectomy for metastatic colorectal cancer. Conversely, those achieving disease-free survival exhibited a striking reduction in NET levels to merely one-quarter of the baseline values 115. The postoperative inflammatory milieu can precipitate systemic immunosuppression 116, engendering the upregulation of various pro-tumorigenic factors and culminating in expedited tumor recurrence. This provokes neutrophils to congregate at sites of trauma, where they release NETs that ensnare CTCs. Najmeh *et al.* adeptly simulated a postoperative inflammatory environment using mouse abdominal sepsis models and found that integrin β1 expression was markedly upregulated under these conditions *in vivo*. β1 integrin mediates CTC adhesion to NETs, further reinforcing the assertion that NETs facilitate tumor metastasis 106. Moreover, Spicer *et al.* made intriguing observations wherein NETs were deposited and seized circulating lung cancer cells in mouse models subjected to cecal ligation and puncture, with liver metastases emerging just 48 h following lung cancer cell injection, with an increase in metastatic load evident 2 weeks later 69. Tohme *et al.* further substantiated the premise that NET formation exacerbates tumor metastasis after surgical stress 115. Ren *et al.* 117 provided compelling evidence that platelets enhance NET-mediated capture of CTCs and consequently propel metastatic progression. NETs play a critical role in promoting cancer metastasis by regulating inflammatory responses.

### 5.2. NETs and pre-metastasis niche

Laminin, a key ECM component, undergoes degradation that disrupts the basement membrane, initiating tumor invasion and metastasis 66. NET-derived proteases, such as NE and MMP-9, remodel laminin and activate integrin α3β1 signaling, promoting cancer cell proliferation 82. Notably, integrin β1 signaling drives the transition from dormancy to metastatic growth 118. Different NET components contribute to metastasis through distinct mechanisms. NE promotes invasion by directly degrading ECM proteins and activating protease cascades 119, while MMP-9 disrupts ECM integrity by degrading type IV collagen in the basement membrane, facilitating tumor cell dissemination 120. Additionally, CG enhances metastasis by activating MMPs and hydrolyzing ECM components, as demonstrated in liver cancer models 55.

NETs may capture CTCs, exposing them to a protein-rich microenvironment that promotes proliferation and metastasis. This interaction could further enhance the ability of CTCs to colonize distant organs 121. Before the actual metastatic process, the primary neoplasm cultivates a microenvironment within a remote organ that fosters cancer dissemination—this phenomenon is termed a “premetastatic niche (PMN)” 122." Castano *et al.* 123 elucidated that in early-stage ovarian cancer, factors derived from ovarian malignancies initially catalyze the formation of NETs and establish PMNs, forging an optimal milieu for subsequent metastasis. Subsequently, NETs act as mediators, through which CTCs amplify the spread of OCs. Wculek *et al.* 124 noted that neutrophils residing within these PMNs enhance subpopulations of malignant cells by releasing leukotrienes, promoting breast cancer dissemination. Owing to the existence of PMNs, carcinoma cells originating from primary tumors usually remain quiescent upon the invasion of other tissues. However, NETs can rouse dormant carcinogenic cells back into active proliferation. Albrengues *et al.* 82 discovered that lipopolysaccharides can awaken dormant cancer cells; however, depletion of neutrophils can negate this effect. The utilization of PAD4 inhibitors or DNases has been shown to diminish both LPS-induced formation of NETs and awakening of cancer cells. They proposed a nuanced mechanism through which NETs rouse these quiescent cells: NE and MMP-9 derived from NETs cleave and remodel laminin, activating the integrin α3β1 signaling pathway and fostering cancer cell proliferation.

Metastasis remains a pivotal factor contributing to poor survival rates in patients with cancer. Dormant cancer cells can remain concealed within PMNs until favorable sites for colonization are identified; this phenomenon is prevalent in breast cancer, prostate carcinoma, and melanoma. Consequently, investigating the intricate relationship between NETs and PMNs is important and offers valuable insights into strategies aimed at curtailing cancer metastasis.

### 5.3. NETs and angiogenesis

To sustain tumor growth, an adequate supply of blood is required 125. Aldabbous *et al.* 126 demonstrated that NETs directly promote angiogenesis. Tumor cells traverse the vascular endothelial barrier, infiltrate the circulation, and colonize distant tissues, facilitating metastasis 127, 128. This process depends on two key factors: cancer cell plasticity and vascular integrity 129. Jiang *et al.* 67 pioneered an *in vitro* co-culture system using human umbilical vein endothelial cells (HUVECs) to investigate the impact of NETs on oncological fluidity. Their findings revealed that NETs downregulated vascular endothelial cadherin, compromised endothelial integrity, and facilitated extravascular infiltration by HCC, augmenting the metastatic potential. Given the tortuous and highly permeable nature of tumor vasculature, it is particularly vulnerable to NET-mediated remodeling. NETs have been shown to enhance endothelial sprouting, tube formation, and vascular permeability 126. McDowell *et al.* 120 discovered that NET formation significantly affects endothelial cell-cell and contacts and enhances vascular permeability, which catalyzed cancer metastasis. The NET-DNA receptor CCDC25, expressed in HUVECs, mediates NET-driven endothelial proliferation, migration, and tubulation. In an *in vitro* rat aortic explant model, NETs promoted endothelial survival and chemotaxis, effects comparable to VEGF stimulation 130. NETs play a role in pathological vascular remodeling in other disease contexts. In proliferative diabetic retinopathy and ischemic retinopathy models, senescent vasculature releases factors that attract neutrophils, triggering NET formation. This process clears dysfunctional endothelial cells and facilitates vessel remodeling, further supporting the role of NET-associated proteases in shaping the tumor vasculature 131. Moreover, MMP-9—an integral component of NETs—has been implicated in promoting angiogenesis, reinforcing the concept that NETs contribute to the vascular adaptations necessary for tumor progression.

### 5.4. NETs and metastasis cascade

In a mouse in situ breast cancer model constructed by Park *et al.* 39 without systemic inflammation, tumors induced by metastatic cell lines recruited a higher proportion of NETs to the primary tumor than those induced by non-metastatic cell lines, and intravenous injection of metastatic cell lines resulted in the deposition of NETs in the lung, which was conducive to the formation of a metastatic microenvironment. One mechanism by which NETs stimulate cancer cell migration is through chemotaxis of NET-DNA to DNA sensors, such as CCDC25. Yang *et al.* 20 found that neutrophils, NETs markers MPO, and CitH3 exist in primary tumors and liver metastatic tumors of patients with breast cancer, and proved that the DNA components of NETs in the liver interact with the transmembrane protein CCDC25, and DNA-CCC25 triggers a cascade of intracellular signaling. The promotion of anticancer antibodies against CCDC25 significantly reduced NET-mediated liver metastasis in breast cancer. Tang *et al.* 107 further expanded upon these findings by investigating 4T1 cells and discovered that ASPP2-induced promotion of cholesterol biosynthesis culminated in NET formation, indicating that de novo cholesterol synthesis plays a crucial role in neutrophil recruitment and the subsequent development of NETs, whereas high levels of cholesterol stimulated CCDC25 expression, a regulatory mechanism requiring deeper exploration.

NETs can also catalyze cancer metastasis through mechanisms distinct from protein interactions, such as eliciting endothelial damage and facilitating EMT. EMT induction augmented the aggressiveness of epithelial carcinoma cells. Co-culturing NETs with cancer cells within a breast cancer milieu revealed downregulation of E-cadherin coupled with upregulation of mesenchymal markers 65, substantiating the notion that NEThe induces gastric cancer cell metastasis via EMT activation, specifically targeting NETs. Zhu *et al.* 132 observed a marked decrease in the levels of E-cadherin in gastric adenocarcinoma cells, concomitant with an elevation in mesenchymal markers. In a comprehensive analysis conducted by Martins-Cardoso *et al.* 65, the influence of NETs on the phenotypic characteristics of human breast cancer cells was examined using a sophisticated breast cancer model. Their findings revealed that exposure to NETs effectively transformed the epithelial morphology of MCF-7 cells into a mesenchymal phenotype, accompanied by significant alterations in the EMT attributes. There was an upregulation of N-cadherin and fibronectin expression, whereas E-cadherin levels were inhibited. These EMT modifications may not only correlate with morphological changes at the cellular level, but can also augment cellular migratory capabilities. Such investigations highlight that NETs possess the potential to facilitate cancer metastasis through intricate protein interactions or by orchestrating metastatic cascades such as tumor-derived EMT processes.

## 6. NETs and cancer-related thrombosis

**Figure 6:**
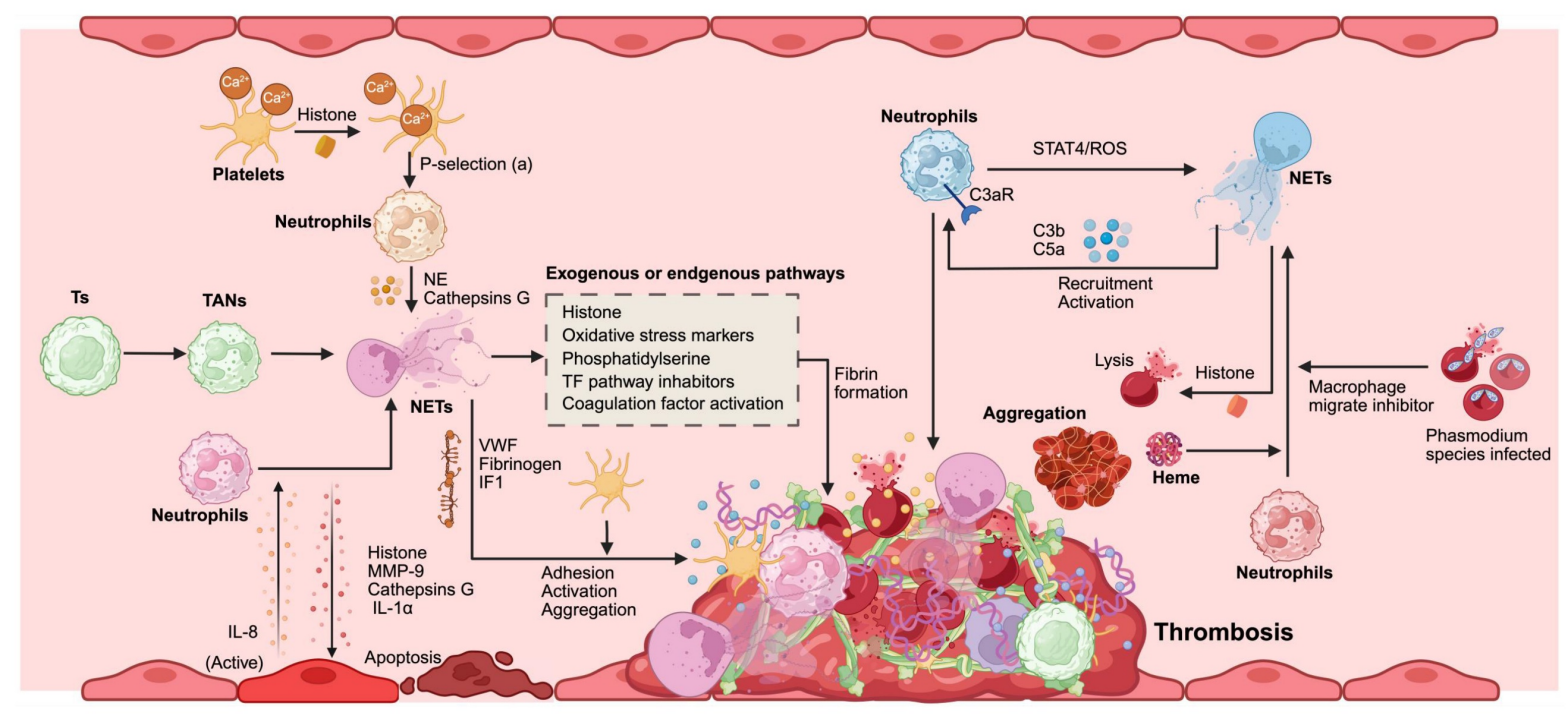
** NETs promote coagulation and cancer-associated thrombosis.** Platelets increase intracellular calcium due to histone activation, which triggers neutrophil activation through P-selectin signaling. Tumor cells (Ts) recruit tumor-associated neutrophils (TANs) and induce their transformation into NETs. Activated endothelial cells secrete IL-8, promoting NET formation. Depending on the microenvironment, NET components can activate or induce apoptosis in endothelial cells. Erythrocytes infected with Phasmodium species and free heme participate directly or indirectly in the conversion of neutrophils into NETs. NETs contribute to thrombosis through several mechanisms. Initially, they promote platelet adhesion, activation, and aggregation through interactions with von Willebrand factor (VWF), fibrinogen, and intermediate filament 1(IF1). Both exogenous and endogenous pathways involving histones, oxidative stress markers, phosphatidylserine, and tissue factor (TF) pathway inhibitors facilitate NET-mediated fibrin formation. Additionally, a positive feedback loop involving neutrophils exists: complement activation through C3a receptor (C3aR), signal STAT4/ROS signaling promotes NET formation. Concurrently, C3b and C5a mediate further neutrophil recruitment and activation. These processes collectively contribute to erythrocyte lysis and aggregation, crucial for thrombus formation in cancer-related contexts. Abbreviations: TANs, tumor-associated neutrophils; Ts, Tumor cells; VWF, von Willebrand factor; IF1, intermediate filament 1; TF, tissue factor; C3aR, C3a receptor; ROS, reactive oxygen species. *Created with BioRender.com.

Demers *et al.* meticulously examined the intricate relationship between NETs and tumor-associated thrombosis, ultimately concluding that after chemotherapy for tumors, the liberation of NETs alongside the thrombin-antithrombin complex precipitates a process known as NETosis. This cascade exacerbates both inflammation and coagulation processes; these inflammatory responses activate neutrophils, further promoting the generation of additional NETs. Hence, NETs are believed to function as potent enhancers of coagulation in malignancy-related thrombosis 133. Jung *et al.* investigated whether NET formation catalyzes endogenous plasma thrombin production. Their experiments revealed that pancreatic cancer cells and their conditioned media can induce NETs formation, significantly augmenting their ability to produce normal endogenous plasma thrombin 52. Boone *et al.*, through their findings, corroborated that thrombosis associated with pancreatic carcinoma is intrinsically linked to NET formation. In an experimental cohort involving mice burdened with tumors, but genetically modified to lack PAD4, an essential enzyme for NETs creation, it was observed that these subjects generated no NETs. Moreover, hydroxychloroquine (an inhibitor of NETs formation) has demonstrated efficacy in diminishing platelet aggregation, curtailing circulating tissue factors, and mitigating blood hypercoagulability. Clinical data have revealed a striking reduction in venous thromboembolism rates among patients with pancreatic cancer treated with hydroxychloroquine after chemotherapy, from 30% to 9.1% 134. Seo *et al.* elucidated the mechanism underlying portal vein thrombosis in patients with liver cancer through clinical evaluation involving 177 hepatitis carrier cases, including 77 afflicted individuals with portal vein thrombosis. They compared them with 48 healthy controls, in which markers indicative of NETs formation (such as DNA-histone complexes, double-stranded DNA fragments, and NE levels) exhibited significantly elevated concentrations relative to those found in healthy subjects. This finding substantiates contact system activation as a novel mechanistic pathway that contributes to thrombus development 135. NETs represent a pivotal mechanism of the innate immune response within the body and play an integral role in the intricate process of thrombosis. The distinct reticular architecture of NETs serves as a scaffold for thrombus formation. NETs facilitate thrombosis through dynamic interactions with coagulation factors, complement proteins, platelets, endothelial cells, and erythrocytes** (Figure 6)**.

### 6.1. NETs and coagulation cascade

NETs possess the intriguing capability to initiate the coagulation cascade directly. DNA embedded within NETs enhances the activity of serine proteases, whereas histones are implicated in augmenting thrombin production. Paradoxically, intact NETs cannot activate coagulation in vitro 136. This phenomenon may be because of the selective exposure of tissue factors present within NET structures 137. Furthermore, negatively charged NETs can engage directly with and activate endogenous promoters of the coagulation pathway, notably the coagulation factor XII (FⅫ). NET-induced thrombin generation was markedly diminished in plasma derived from mice deficient in either F-XI or F-XII 138. Wang *et al.* 139 also showed that neutrophil microparticles can attach to NETs through interactions between histones and phosphatidylserine, instigating thrombin production via endogenous mechanisms of coagulation. In addition, NE and cathepsin G within NETs play a significant role in facilitating fibrin formation by degrading tissue factor pathway inhibitors 9. The processes of fibrin formation and deposition transpire upon activation by either exogenous or endogenous coagulation pathways on NETs structures; however, fibrin clots intertwined with NETs exhibit enhanced resistance to plasminase. This characteristic may constitute a pivotal challenge for thromboembolic diseases 137.

### 6.2. NETs and component systems

The initial indication of the impact of the complement system on NETs emerged from observations that neutrophils derived from complement 3 (C3) or complement C3a receptor(C3aR) knockout mice exhibited a marked inability to form NETs 140. Guglietta *et al.* 141 elucidated that the release of LPS-induced NETs is contingent on the activation of the complement cascade and the subsequent upregulation of C3aR on neutrophil surfaces, which fosters coagulation. The complement component C5a plays a pivotal role in recruiting and activating neutrophils, and serves as a frequently used irritant in prompting NETosis. Chen *et al.* 142 demonstrated that C5a amplified ROS production by inhibiting STAT4 activity during NETosis, stimulating NETosis, and facilitating arterial thrombosis formation. The released NETs create an environment that is conducive to complement activation. The complement component C1q deposited onto these structures stabilizes their integrity by impeding the action of deoxyribonuclease I 143. In contrast, it enhances macrophage activation through the high expression of the C1q receptor induced by NETs 144. Furthermore, complement factor H has been shown to suppress PMA-induced NET formation while diminishing the deposition of complement fragment C3b on these traps 145, 146. In addition, proteins such as MPO residing within NETs actively participate in complement activation processes, cleaving complement component C5 into its active fragments, and cathepsin G and neutrophil elastase target C3 137. This intricate interplay further highlights the correlation between thrombosis onset, NET release dynamics, and the activation of complementary pathways.

### 6.3. NETs and platelets

After perfusion of whole blood with NETs, one can observe that NETs serve as scaffolding structures, facilitating the adsorption of platelet aggregation 147. Activated platelets express P-selectin, which interacts with P-selectin glycoprotein ligand 1 present on the surface of neutrophils, promoting the formation of NETs 148, 149. Furthermore, platelets release the HMGB1 protein, a crucial factor that enhances NET formation. HMGB1 elicits the release of NETs by activating neutrophils via multiple receptors, such as TLR2 and TLR4 150. Experiments have demonstrated that the interaction between platelets and NETs is contingent on histones; purified histones stimulate calcium influx into platelets *in vitro*, inducing their activation 9. In addition, NE and cathepsin G within NETs can promote platelet aggregation by activating platelet surface receptors 151. The interplay between NETs and platelets may also be mediated by various adhesion molecules, including von Willebrand factor (VWF) and fibrinogen 151. In summary, a significant interaction exists between activated platelets and NETs. Activated platelets trigger the release of NETs, which further amplify platelet adhesion, activation, and aggregation, ultimately engaging in procoagulant activity that creates a positive feedback loop that enhances coagulation.

### 6.4. NETs and endothelial cells

Preservation of the integrity of endothelial architecture and its multifaceted functions is paramount for sustaining vascular homeostasis; however, endothelial injury is a pivotal contributor to thrombosis. Co-culture of endothelial cells and neutrophils demonstrated that activated endothelial cells can precipitate the release of NETs, a mechanism intricately reliant on IL-8 secreted during the activation of these cells 40. Concurrently, components associated with NETs can induce both the activation and apoptosis of endothelial cells, and this reciprocal feedback loop significantly exacerbates thrombotic processes. Histone proteins exhibit pronounced affinity for phospholipids, facilitating their binding to cellular membranes and inducing calcium ion influx. This cascade further amplifies endothelial cell activation while promoting exocytosis in Weibel-Palade bodies 152. MMP-9 present within NETs may induce damage to endothelial cells through an activation pathway involving MMP-2, participating in pathological phenomena such as thrombosis 153, 154. Folco *et al.* 155 reported that NETs activate endothelial cells to express adhesion molecules via synergistic interactions between IL-1α and cathepsin G, culminating in localized vascular inflammation accompanied by thrombosis. In parallel with the research conducted by Blanco *et al.* 156, it was revealed that small RNAs encapsulated within NETs could be internalized by endothelial cells, triggering a type I interferon response, an event believed to mediate vascular injury and advance atherosclerotic processes. VWF, a glycoprotein produced by activated platelets or directly from endothelium-derived sources, plays an essential role in hemostasis. The release of NETs from neutrophils has been shown to elevate VWF concentrations while fostering polymer formation through mechanisms such as oxidation, citrullination, hydrolysis mediated by ADAMTS13, or competitive interactions with VWF itself 157. Henceforth, we observed how NETs serve not merely as agents, but help to perpetuate dysfunction within the endothelium associated with thrombus formation.

### 6.5. NETs and red blood cells

Red blood cells are intrinsically linked to thrombosis and can facilitate coagulation through a myriad of mechanisms, including alterations in blood viscosity, exposure to phosphatidylserine, and adhesion to platelets 158. Immunohistochemical staining of murine models of deep vein thrombosis and human coronary artery thrombi revealed pronounced accumulation of red blood cells surrounding NETs 159, 160. This observation implies that the interplay between NETs and blood cells may play a pivotal role in thrombosis progression. Kordbacheh *et al.* 161 discovered that extracellular histones can provoke erythrocyte aggregation and lysis; moreover, heme liberated from ruptured erythrocytes has been shown to instigate NETs formation 162. Red blood cells infected with Plasmodium species can stimulate neutrophils to release NETs by discharging macrophage migration inhibitors and heme substances 162, 163. However, the exact mechanisms underlying the interaction between blood cells and NETs require further investigation.

## 7. NET interaction with immune cells in cancer

**Figure 7:**
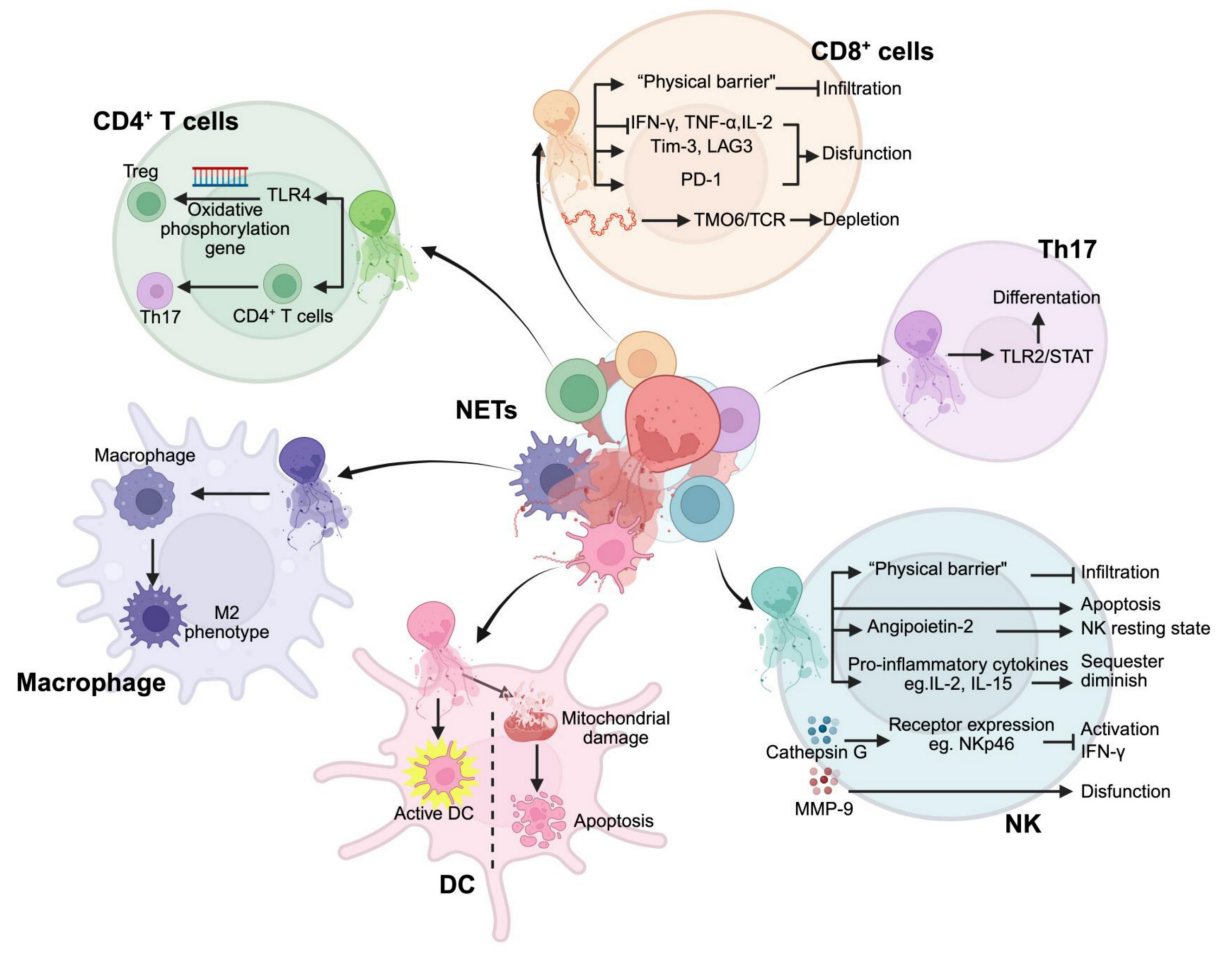
** NETs regulate the functions of immune cells in the tumor microenvironment.** NETs modulate multiple immune cell populations through distinct mechanisms: In CD4+ T cells, NETs trigger TLR4-mediated oxidative phosphorylation gene regulation, affecting both regulatory T cells (Treg) and Th17 differentiation. CD8+ T cells respond to NETs through multiple pathways: forming physical barriers that inhibit infiltration, suppressing cytokine production (IFN-γ, TNF-α, IL-2), inducing functional proteins (Tim-3, LAG3, PD-1), and causing T cell depletion via TMO6/TCR signaling. NETs promote Th17 cell differentiation through TLR2/STAT pathway activation. In the NK cell compartment, NETs create physical barriers preventing infiltration, while inducing apoptosis and NK cell dysfunction through Angiopoietin-2 and inflammatory cytokines (IL-2, IL-15). NETs-derived cathepsin G and MMP-9 regulate NK cell receptor expression (e.g., NKp46) affecting IFN-γ production and function. Dendritic cells (DCs) respond to NETs through two opposite pathways: activation of DCs and mitochondrial damage-induced apoptosis. Macrophages exposed to NETs undergo polarization toward an M2 phenotype. Abbreviations: Treg; regulatory T cells; NK, natural killer cells; DC, dendritic cells. *Created with BioRender.com.

The immunosuppressive TME is a key driver of tumor immune evasion 164. Persistent inflammatory stimuli, chemokines, and metabolic factors drive neutrophil chemotaxis toward tumor tissues, where they actively reshape the TME. This remodeling fosters the infiltration of immunosuppressive cells, particularly myeloid-derived suppressor cells (MDSCs) and regulatory T cells (Tregs), thereby reinforcing mechanisms of tumor recurrence and metastasis 165, 166. As a key player in this process, NETs not only sustain an immunosuppressive niche but also disrupt anti-tumor immune responses, positioning them as critical regulators of immune escape **(Figure 7)**. The emergence of immune checkpoint inhibitors, such as anti-PD-1/PD-L1 therapies, has transformed cancer treatment. However, accumulating evidence suggests that NETs may limit their efficacy. Studies indicate that targeting NETs can enhance the therapeutic effects of anti-PD-1 therapy, as demonstrated in MC-38 colon cancer models 167, while NET-based prognostic models show promise in predicting immunotherapy responses 168. Furthermore, inhibiting NET formation has been linked to improved anti-PD-1 treatment outcomes in liver, pancreatic, and breast cancers 58, 169. These findings highlight NETs as key mediators of tumor progression through immune suppression, warranting further investigation into their role as therapeutic targets.

Despite these insights, the mechanistic underpinnings of NET-driven immune resistance remain poorly defined. How NETs modulate T cell exhaustion, suppress antigen presentation, or interact with metabolic constraints within the TME is not fully understood. This gap underscores the need for refined therapeutic strategies that selectively dismantle NET-mediated immunosuppression while preserving their potential role in host defense. Future research should aim to dissect these molecular pathways and explore the synergistic potential of NET-targeting approaches in combination with immunotherapy, ultimately paving the way for more effective and durable cancer treatments.

### 7.1. NETs and immunosuppressive cells

A multitude of immunosuppressive cells exists within the TME, including MDSCs, Tregs, TAMs, and Th17 cells. NETs can modulate the expression of genes linked to oxidative phosphorylation in naïve CD4+ T cells via TLR4 signaling, inducing their differentiation into Tregs and facilitating the progression from nonalcoholic steatohepatitis to hepatocellular carcinoma. In addition, NETs have been shown to enhance the infiltration of Tregs and support the local advancement and metastasis of breast cancer 170. According to research conducted by Yu *et al.* 171, NETs can mediate macrophage polarization toward an M2 phenotype, further advancing gastric cancer development. Combined with this phenomenon, they collectively promote invasion and migration of the A549 cell lines 172. While there is currently a lack of reports detailing the interplay between NETs and Th17 cells within the tumoral context, recent investigations suggest that NETs may stimulate Th17 cell differentiation through activation of the TLR2-STAT signaling pathway 173. Furthermore, these traps have been implicated in driving CD4+ T cell transformation into the Th17 phenotype, specifically in acute lung injury 174. Taken together, these results demonstrate that NETs critically contribute to tumor advancement through enhancing the activity of diverse immunosuppressive cell populations.

### 7.2. NETs and dendritic cells

Dendritic cells (DCs) are the principal antigen-presenting entities within the body and play a pivotal role in curbing tumor progression by eliciting an antigen-specific adaptive immune response 175. Numerous studies have shown that NETs possess the capability to activate DCs while simultaneously delivering pertinent antigens that foster specific immunity, impeding the advancement of NPM-mutant myeloid leukemia. Conversely, NETs induce DC apoptosis by inducing mitochondrial damage 176, 177. In collection, these investigations underscore the dualistic nature of NETs' influence on DCs; they not only facilitate antigen presentation and prime the body's immune response, but may also precipitate apoptotic pathways within DC, ultimately hindering adaptive immunity. Factors such as NET composition and the tumor microenvironment likely dictate whether NETs enhance antigen presentation or induce DC apoptosis. This complexity underscores the need for targeted strategies that mitigate NET-induced immunosuppression while preserving their immunostimulatory potential.

### 7.3. NETs and CD8+ T cells

Dysfunction of CD8+ T cells is intricately linked to tumorigenesis, progression, and evasion of the disease 178. NETs are strategically positioned around tumor cells, acting as a formidable "physical barrier" that facilitates tumor advancement by constraining the infiltration of CD8+ T cells and NK cells. Beyond merely inhibiting cellular infiltration, NETs profoundly induce CD8+ T cells in various tumor models. For instance, these structures diminish the secretion of critical effector cytokines from CD8+ T cells, such as interferon-gamma (IFN-γ), TNF-α, and IL-2, while simultaneously upregulating immune checkpoint molecules, such as Tim-3 and LAG3. This indicates that NETs play a pivotal role in lung cancer progression by inducing CD8+ T cell dysfunction 179. Moreover, components such as PD-L1 present within NETs have been shown to inhibit the functionality of CD8+ T cells by engaging PD-1 receptors on the surface membranes of these lymphocytes 83. In liver cancer, DNA fragments derived from NETs obstruct both T cell receptor (TCR) signaling and activation pathways, including NF-kB, by binding to TMCO6 on the membranes of CD8+ T cells. This interaction precipitates a depletion effect on these crucial immune players, while concurrently promoting malignant growth 180. Research focusing on breast cancer has revealed that targeting NETs can effectively curtail T cell infiltration and significantly enhance the therapeutic efficacy of anti-PD-1 monoclonal antibodies 58. Besides breast carcinoma, numerous studies have independently demonstrated that disrupting NET formation augments the effectiveness of anti-PD-1 therapy in various malignancies, including colorectal cancer, lung cancer, and hepatocellular carcinoma 181, 182. Collectively, these investigations underscore how NETs compromise CD8+ T cell function and hinder the effective action of immune checkpoint inhibitors in tumors. Therefore, strategies aimed at targeting NETs hold promise for revitalizing CD8+ T cell efficacy and improving immunotherapeutic outcomes.

### 7.4. NETs and NK cells

NETs can impede the anti-tumor efficacy of NK cells via a multitude of mechanisms. First, as previously indicated, NK cells are hindered from effectively engaging tumor cells because of the obstruction posed by NETs. Furthermore, NETs can degrade proinflammatory cytokines that are imperative for the activation, proliferation, and functional performance of NK cells, such as IL-2 and IL-15 183, 184. Consequently, NETs may indirectly suppress the anti-tumor properties of NK cells by sequestering and diminishing critical proinflammatory signals. In addition, NETs have been shown to directly induce apoptosis in NK cells 75. CG is a vital component of NETs; it can undermine NK cell activation as well as IFN-γ release and degranulation processes by downregulating the expression of activated receptors, such as NKp46 185. Moreover, MMP-9 abundance within NETs may further contribute to the dysfunctional behavior of NK cells, facilitating immune evasion by malignant tumor cells 76. NETs interact with various elements within the TME to modulate NK cell function. For instance, in gastric cancer models, NETs have been observed to elevate angiopoietin-2 levels within the TME, which is intricately linked to the maintenance of intact resting states among NK cell populations 186, suggesting that NETs might indirectly influence the anti-tumor activity of these immune effector cells via the regulation of TME constituents. Cheng *et al.* 77 discovered that inhibiting NET formation significantly enhances both functionally active capacities in liver cancer-associated NK cells while simultaneously impeding disease progression, underscoring the potential for future exploration of interactions between NET profiles and NK cell dynamics to optimize therapeutic strategies focused on enhancing cellular immunotherapy against tumors.

In conclusion, the presence of NETs significantly amplified the infiltration and functionality of immunosuppressive cells while markedly undermining the activities of NK and CD8+ T cells. This disruption leads to the breakdown of immune homeostasis within the body, promoting immune evasion by tumor cells and facilitating disease progression. These findings highlight the potential of NETs as novel therapeutic targets in tumor immunotherapy. Within the rapidly advancing landscape of cancer immunotherapy, strategic targeting of NETs may greatly enhance the efficacy of immune checkpoint inhibitors in various malignancies. This approach offers innovative strategies to address the challenges posed by suboptimal treatment response rates and emerging resistance mechanisms. We posit that a thorough investigation of the specific mechanisms used by NETs within the tumor microenvironment, along with the development of effective and targeted interventions against NETs, will be crucial for evaluating their synergistic effects with other immunotherapeutic modalities. This trajectory promises to propel continued innovation and advancement in the field of tumor immunotherapy.

## 8. Target NETs for cancer treatment

### 8.1. DNA inhibitor

Recombinant human DNase I is a multifaceted therapeutic agent used to manage bronchiectasis and lung abscesses. Its myriad functions include the absorption of nucleotide nutrients, modulation of biofilm formation, facilitation of pathogen invasion, degradation of DNA matrices, and regulation of immune responses, among others 187. However, their clinical application is hindered by the short half-life of the endonuclease enzymes. While the U.S. The Food and Drug Administration has sanctioned an inhalable formulation, pulmozyme, indicated for cystic fibrosis treatment, but the ability of such aerosolized preparations to penetrate the systemic circulation remains limited. This results in suboptimal efficacy when addressing NETs beyond the pulmonary or vascular confines. Moreover, this approach exhibits insufficient targeting capabilities and poses certain risks to overall health 188. In an innovative stride forward, Xia *et al.* 189 engineered an adeno-associated virus (AAV) gene therapy vector specifically designed for the hepatic expression of DNase I. Their research demonstrated that AAV-mediated delivery of DNase I significantly inhibited liver metastasis from colorectal cancer in murine models following a single intravenous administration. These findings suggested that AAV-mediated DNase I treatment is a safe and effective therapeutic strategy. Consequently, DNase I inhibitors exhibit substantial potential as targeted therapies for NETs and warrant further investigation to elucidate their clinical applications.

### 8.2. PAD4 inhibitors

PAD4 is a pivotal molecule in the intricate formation of NETs and exerts regulatory control over the citrullination of histone proteins. This process culminates in the disruption of chromatin DNA within neutrophil nuclei, facilitating the subsequent generation of NETs 190. Inhibition of PAD4 has demonstrated efficacy in diminishing circulating levels of NETs and abolishing cancer-induced NET formation. Studies involving PAD4-deficient murine models have revealed pronounced inhibition of NETs formation, which concurrently impedes tumor progression and prolongs the survival span of these subjects 191. Research indicates that targeting PAD4 through its inhibition is an exceptional strategy for curtailing NET activity; among the potential agents are small-molecule inhibitors such as chloramidine, an irreversible inhibitor, and GSK484, which offers reversible inhibitory action against PAD4 192. Nevertheless, challenges remain owing to the rapid metabolic degradation and suboptimal oral bioavailability of the existing PAD4 inhibitors. Considering these drawbacks, recent studies have focused on innovative nanodelivery systems for PAD4 inhibitor development. These include the exploration of AuNPs used as nanomaterials 193, covalent linkages with chitosan to fabricate oxidative stress-responsive nanosystems 194, and liposomal carriers engineered for targeted delivery 195.

### 8.3. Inhibiting inflammatory factors

Another approach for inhibiting NET formation involves targeting upstream mediators, a domain that has garnered considerable attention in studies of inflammation-related factors. CXCR1/2, a G-protein-coupled transmembrane receptor on the surface of neutrophils, is a critical mediator of nuclear chemotactic recruitment of neutrophils 196. IL-8 is a pivotal and efficacious agent that facilitates chemotaxis by interacting with the aforementioned receptor. Recent reports have indicated that the synergistic application of IL-8 monoclonal antibodies with CXCR1/2 inhibitors demonstrates promising efficacy and has the potential to be an innovative therapeutic strategy for targeting NETs 197. Several pharmacological agents are under development for repurposing. For instance, anti-interleukin-17 antibodies used in psoriasis treatment may possess the capacity to modulate neutrophil recruitment while simultaneously inhibiting NET formation 57. In addition, complement C5a receptor 1 inhibitors have received approval for their use in combating antineutrophil-associated small vessel vasculitis 198.

### 8.4. Traditional Chinese medicine

Tang *et al.* 39 elucidated that lipid-lowering agents, such as simvastatin, possess the capacity to modulate cholesterol synthesis and indirectly influence the expression of CCDC25, curbing the formation of NETs. Berberine, a naturally occurring compound prevalent in traditional Chinese medicine, is recognized for its lipid-lowering properties and its regulatory effects on NET formation. Dihydrotanshinone I (DHT), the principal active constituent of Salvia miltiorrhiza, inhibits the proliferation of breast cancer cells. Zhao *et al.* 199 posited that DHT could diminish the expression of tissue inhibitor of metalloproteinase-1 (TIMP1), which is generated during NET formation, consequently mitigating NET production. Pan *et al.* 200 discovered that Huangqin Decoction effectively reduced the levels of IL-1 and MMP-9 in a murine model of colorectal cancer while simultaneously inhibiting PAD4 expression, which impeded the progression of colorectal carcinoma by hindering NETs generation. Haute *et al.* 201 demonstrated the ability of octyl gallate to suppress the release of ROS and regulate lipopolysaccharide-induced neutrophil apoptosis. However, the intricate mechanisms underlying its inhibitory effects on NETs production remain unclear. The bioactive constituents derived from single herbs and compounds in traditional Chinese medicine demonstrate the potential to regulate NETs by modulating factors such as ROS, PAD4, and TIMP1. Nonetheless, current research on traditional Chinese medicine remains nascent, with insufficient clinical trials. There is an imperative need for further investigation of the precise mechanistic pathways associated with the components found in traditional Chinese medicines to more effectively underscore their therapeutic value against targeted NETs.

### 8.5. Antibiotics

Antibiotics represent a distinct class of secondary metabolites synthesized by microorganisms, higher plants, and animals that confer resistance against pathogens. Bystrzycka *et al.* 202 investigated neutrophils treated with chloramphenicol and azithromycin and found a significant reduction in NET formation. Furthermore, azithromycin administration appears to diminish the likelihood of respiratory disease outbreaks. Although agents such as gentamicin have similar inhibitory effects on NET formation, cefotaxime demonstrates no therapeutic efficacy. Consequently, it is imperative to conduct more in-depth research on the judicious application of antibiotics targeting NETs to better understand their implications and potential benefits.

### 8.6. Nanodrugs for targeting NETs

As mentioned, various strategies have been explored to inhibit NETs and suppress tumor metastasis **(Supplementary Table 1)**, yet clinical translation faces major challenges. Many inhibitors have failed due to low oral bioavailability and poor targeting. Moreover, NET inhibition may impair neutrophil-mediated pathogen defense, increasing infection and carcinogenesis risks, as seen with PAD4 inhibitors and histone citrullination blockade 203. NET degradation may also release DNA-bound proteins like CitH3 and MPO, triggering systemic inflammation 204. Thus, minimizing the adverse effects of NET-targeted therapy remains crucial. Combining NET inhibitors with antibiotics or antivirals may enhance efficacy 192, but optimizing their potency and specificity remains a key challenge.

Nanotechnology has addressed major limitations of non-specific chemotherapy by enhancing drug circulation, enabling precise tumor targeting, and minimizing toxicity to healthy cells 205 **(Supplementary Table 1)**. For example, Yin *et al.* 206 developed a smart nanocarrier (mP-NPs-DNase/PTX) targeting tumor-associated NETs, comprising a paclitaxel (PTX) prodrug nanoparticle core and a PLL-based DNase I shell, cleavable by MMP-9. Upon tumor accumulation, MMP-9 triggers DNase I release to degrade NETs, exposing a cell-penetrating peptide that facilitates tumor cell uptake. Intracellularly, high glutathione levels induce PTX release, exerting cytotoxic effects. Beyond its role as a therapeutic agent, nanomedicine can also enhance adoptive NK cell therapy. An injectable hydrogel rapidly forms an adhesive gel, inhibits immunosuppressive cell infiltration by neutralizing tumor acidity, and degrades NETs by releasing pH-responsive DNase I 77. This approach enhances NK cell infiltration and reduces post-surgical HCC recurrence with minimal systemic toxicity 77.

However, TME, characterized by features such as hypoxia, acidity, and elevated interstitial fluid pressure, presents significant challenges that impede the delivery and efficacy of nanomedicines 207, 208. Nanomedicines can interact with neutrophils and potentially induce NETosis, a process that may have dual effects: either promoting anti-tumor immune responses 209 or, if uncontrolled, exacerbating tumor progression 210. Therefore, a comprehensive understanding of the molecular mechanisms, transcription factors, and signaling pathways involved in NETosis—and how these can be modulated by nanomedicines—is crucial 209. Despite notable progress, several gaps remain in our understanding of nanomedicine, particularly regarding its interaction with the TME and immune system. Further research is required to establish foundational principles for nanomedicine design, develop advanced material platforms, and create relevant animal models that closely mirror human tumor biology 211. Additionally, in-depth studies on tumor biology, including intratumor heterogeneity and nano-bio interactions, are essential for designing nanomedicines capable of effectively navigating the TME and delivering therapeutic payloads.

## 9. Conclusion and perspectives

NETs have attracted increasing attention in cancer research due to their role in tumor progression. Evidence suggests that NETs facilitate metastasis by modulating the tumor immune microenvironment, vascular system, and premetastatic niche formation. However, some studies indicate that NETs may exhibit tumor-suppressive effects under certain conditions. The functional outcomes of NET-tumor interactions are influenced by neutrophil subtypes, NET composition and formation mechanisms, tumor microenvironment heterogeneity, and experimental conditions. Thus, further elucidation of the determinants shaping NET activity in cancer is of critical importance.

Despite growing research efforts, the precise mechanisms underlying NET-mediated tumor progression remain unclear, limiting their clinical translation. Inhibitors such as chloramidine, recombinant human DNase I, antibiotics, and certain traditional Chinese medicine formulations have demonstrated efficacy in suppressing NET formation, offering potential therapeutic benefits. Additionally, the combination of NET inhibitors with immunosuppressants may represent a novel anti-tumor strategy. Advances in nanotechnology further enable the development of smart drug delivery systems, enhancing targeted therapy while mitigating adverse effects.

Several key knowledge gaps warrant further investigation. First, the extent to which NET formation mechanisms influence their structural composition and biological function remains unresolved. Second, despite the increasing volume of research on NETs since their discovery 18 years ago, the absence of a standardized detection method complicates cross-study comparisons and reproducibility. Third, while substantial progress has been made in understanding NETs in tumor progression, their effects are highly tissue- and context-dependent. Distinct polymorphonuclear neutrophil subpopulations may generate functionally diverse NETs, and variations in the immune microenvironment may modulate their impact. Lastly, interdisciplinary efforts are essential for the development of spatiotemporally controlled drug delivery systems targeting NET-associated cancers, necessitating further clinical validation. In conclusion, a mechanistic dissection of NET-cancer interactions holds significant promise and warrants rigorous investigation to inform future clinical applications.

## Supplementary Material

Supplementary table.
