# Supplementary material for: Targeting neutrophil extracellular traps in cancer progression and metastasis
Source: Theranostics. 2025 Apr 22;15(12):5846–69. doi: 10.7150/thno.111096 (PMC12068306; doi:10.7150/thno.111096)
Supplement: Supplementary file 1 — Supplementary table. [file thnov15p5846s1.pdf]

Supplementary Table 1. Therapeutic Agents Targeting NETs in Cancer

Target molecules	Agents	Mechanism/Results	Cancers	References
**PAD4 inhibitors**				
PAD4	Chloroquine	Reduce NET formation through inhibing TLR9 and PAD4 inhibitory.	Pancreatic cancer	[1]
PAD4	ZD-E-1	Inhibited tumor growth and metastasis by reduce PAD4 activity and NETs formation.	Lung cancer, Breast cancer	[2]
PAD4	BB-Cl-amidine	Decreased NF-κB activation and tumor metastasis.	Breast cancer	[3]
PAD4	GSK484	As immunotherapeutic synergy to inhibite NET-mediated barrier protection.	Breast cancer	[4]
PAD4	GSK484	Inhibit tumor metastasis by deplete NETs.	Hepatocellular carcinoma	[5]
**DNA inhibitors**				
DNA	DNase1	Inhibit NET formation and decrease tumor growth.	Nonalcoholic steatohepatitis to hepatocellular carcinoma	[6]
DNA	DNase 1	Disrupt NET-driven tumor cell capture and metastasis.	Colorectal cancer	[7]
DNA	DNase + PD-1	Reversal of anti-PD-1 blockade resistance through	Colorectal cancer	[8]

		increasing cytotoxicity.		
DNA	AAV-DNase I	Inhibite neutrophil infiltration and NET formation	Colorectal cancer	[9]
DNA	DNase I	Reduce lung metastases.	Breast cancer	[10]
DNA	DNase I	Improve overall radiation response via inhibiting NETs formation.	Bladder cancer	[11]
**Chemokines**				
IL-8、CXCL2	IL-8 antibody、CXCR2 antibody	Promote Diffuse Large B-cell Lymphoma Progression via blocking IL8-CXCR2 axis.	Diffuse large B-cell lymphoma	[12]
IL-8	IL-8 antibody	Regulate the HMGB1/RAGE/IL-8 axis via neutralizing IL-8.	Glioma	[13]
CXCR1/2	Reparixin	As CXCR1/2 inhibitor to inhibite NET-mediated barrier protection.	Breast cancer	[4]
IL-17	IL17/IL17R antibodies	Increase immune checkpoint blockade sensitivity via IL17/IL17R neutralization.	Pancreatic cancer	[14]
**TCM**				
ROS	Danshen(Sal B and DHT I)	Inhibite NETs formation via blocking the activity of MPO and NADPH.	Gastric cancer	[15]
Related to the deactivation of PAD4	Huang Qin Decoction	Inhibits the initiation of colitis associated carcinogenesis by	Colorectal cancer	[16]

		decreasing NETs formation.		
NADPH/ROS-NETs signalling	Kaempferol	Decreased NET formation and tumour metastasis via suppressing ROS production.	Breast Cancer	[17]
**Nanodrugs**				
DNA	mP-NPs-DNase/PTX	Degrade NETS structure.	Non-small cell lung cancer and breast cancer	[18]
DNA	GODM-gel	Buffer tumor acidity and degrade NETs to enhance NK cell cytotoxic function.	Hepatocellular carcinoma	[19]
DNA	PLGA-PD-DNase 1	Inhibit tumor metastasis by degrading NETs structure.	Lung metastasis of breast cancer	[10]
DNA	PR@DNase I-PLGA@Gel	Wreck NETs and inhibit lung metastasis	Lung cancer	[20]
DNA	PAAP/DNase 1	Induce tumor-cell apoptosis and decompose NETS-DNA to prevent NETs-induced tumor metastasis to the liver.	Liver metastasis in CT26 colon cancer and 4TI breast cancer	[21]
DNA	DNase 1+AuPB/mPDA shell	Degrade NETs to expose tumors to immune cells and prevent circulating tumor cells capture.	Colorectal cancer	[7]

NETosis	ZD-E-I	Reduce the growth and metastasis of tumors by Inhibiting histone-3 citrullination and NETs.	Solid tumorst	[2]
NETosis	LA/DOX NPS	Inhibit NETs generation by blocking the NF-kB signaling pathway and P-SEL.	Breast cancer	[22]

1. Ivey AD, Matthew Fagan B, Murthy P, et al. Chloroquine reduces neutrophil extracellular trap (NET) formation through inhibition of peptidyl arginine deiminase 4 (PAD4). *Clin Exp Immunol*. Mar 24 2023;211(3):239-247. doi:10.1093/cei/uxad005
2. Zhu D, Lu Y, Gui L, et al. Self-assembling, pH-responsive nanoflowers for inhibiting PAD4 and neutrophil extracellular trap formation and improving the tumor immune microenvironment. *Acta Pharm Sin B*. May 2022;12(5):2592-2608. doi:10.1016/j.apsb.2021.11.006
3. Zhu B, Zhang X, Sun S, Fu Y, Xie L, Ai P. NF-κB and neutrophil extracellular traps cooperate to promote breast cancer progression and metastasis. *Exp Cell Res*. Aug 15 2021;405(2):112707. doi:10.1016/j.yexcr.2021.112707
4. Teijeira Á, Garasa S, Gato M, et al. CXCR1 and CXCR2 Chemokine Receptor Agonists Produced by Tumors Induce Neutrophil Extracellular Traps that Interfere with Immune Cytotoxicity. *Immunity*. May 19 2020;52(5):856-871.e8. doi:10.1016/j.immuni.2020.03.001
5. Jiang ZZ, Peng ZP, Liu XC, et al. Neutrophil extracellular traps induce tumor metastasis through dual effects on cancer and endothelial cells. *Oncoimmunology*. 2022;11(1):2052418. doi:10.1080/2162402x.2022.2052418
6. van der Windt DJ, Sud V, Zhang H, et al. Neutrophil extracellular traps promote inflammation and development of hepatocellular carcinoma in nonalcoholic steatohepatitis. *Hepatology*. Oct 2018;68(4):1347-1360. doi:10.1002/hep.29914
7. Chen J, Hou S, Liang Q, et al. Localized Degradation of Neutrophil Extracellular Traps by Photoregulated Enzyme Delivery for Cancer Immunotherapy and Metastasis Suppression. *ACS Nano*. Feb 22 2022;16(2):2585-2597. doi:10.1021/acsnano.1c09318
8. Zhang H, Wang Y, Onuma A, et al. Neutrophils Extracellular Traps Inhibition Improves PD-1 Blockade Immunotherapy in Colorectal Cancer. *Cancers (Basel)*. Oct 23 2021;13(21)doi:10.3390/cancers13215333
9. Xia Y, He J, Zhang H, et al. AAV-mediated gene transfer of DNase I in the liver of mice with colorectal cancer reduces liver metastasis and restores local innate and

adaptive immune response. *Mol Oncol*. Nov 2020;14(11):2920-2935. doi:10.1002/1878-0261.12787
10. Park J, Wysocki RW, Amoozgar Z, et al. Cancer cells induce metastasis-supporting neutrophil extracellular DNA traps. *Sci Transl Med*. Oct 19 2016;8(361):361ra138. doi:10.1126/scitranslmed.aag1711
11. Shinde-Jadhav S, Mansure JJ, Rayes RF, et al. Role of neutrophil extracellular traps in radiation resistance of invasive bladder cancer. *Nat Commun*. May 13 2021;12(1):2776. doi:10.1038/s41467-021-23086-z
12. Nie M, Yang L, Bi X, et al. Neutrophil Extracellular Traps Induced by IL8 Promote Diffuse Large B-cell Lymphoma Progression via the TLR9 Signaling. *Clin Cancer Res*. Mar 15 2019;25(6):1867-1879. doi:10.1158/1078-0432.Ccr-18-1226
13. Zha C, Meng X, Li L, et al. Neutrophil extracellular traps mediate the crosstalk between glioma progression and the tumor microenvironment via the HMGB1/RAGE/IL-8 axis. *Cancer Biol Med*. Feb 15 2020;17(1):154-168. doi:10.20892/j.issn.2095-3941.2019.0353
14. Zhang Y, Chandra V, Riquelme Sanchez E, et al. Interleukin-17-induced neutrophil extracellular traps mediate resistance to checkpoint blockade in pancreatic cancer. *J Exp Med*. Dec 7 2020;217(12)doi:10.1084/jem.20190354
15. Tao L, Xu M, Dai X, et al. Polypharmacological Profiles Underlying the Antitumor Property of Salvia miltiorrhiza Root (Danshen) Interfering with NOX-Dependent Neutrophil Extracellular Traps. *Oxid Med Cell Longev*. 2018;2018:4908328. doi:10.1155/2018/4908328
16. Pan Z, Xie X, Chen Y, et al. Huang Qin Decoction inhibits the initiation of experimental colitis associated carcinogenesis by controlling the PAD4 dependent NETs. *Phytomedicine*. Dec 2022;107:154454. doi:10.1016/j.phymed.2022.154454
17. Zeng J, Xu H, Fan PZ, et al. Kaempferol blocks neutrophil extracellular traps formation and reduces tumour metastasis by inhibiting ROS-PAD4 pathway. *J Cell Mol Med*. Jul 2020;24(13):7590-7599. doi:10.1111/jcmm.15394
18. Yin H, Lu H, Xiong Y, et al. Tumor-Associated Neutrophil Extracellular Traps Regulating Nanocarrier-Enhanced Inhibition of Malignant Tumor Growth and Distant Metastasis. *ACS Appl Mater Interfaces*. Dec 22 2021;13(50):59683-59694. doi:10.1021/acsami.1c18660
19. Cheng Y, Gong Y, Chen X, et al. Injectable adhesive hemostatic gel with tumor acidity neutralizer and neutrophil extracellular traps lyase for enhancing adoptive NK cell therapy prevents post-resection recurrence of hepatocellular carcinoma. *Biomaterials*. May 2022;284:121506. doi:10.1016/j.biomaterials.2022.121506
20. Zhou H, Zhu C, Zhao Q, et al. Wrecking neutrophil extracellular traps and antagonizing cancer-associated neurotransmitters by interpenetrating network hydrogels prevent postsurgical cancer relapse and metastases. *Bioact Mater*. Sep 2024;39:14-24. doi:10.1016/j.bioactmat.2024.05.022
21. Lianghan Zhu ZL, Ning Liu, Honghao Sun, Yixin Wang, Minjie Sun. Dynamically Deformable Protein Delivery Strategy Disassembles Neutrophil Extracellular Traps to Prevent Liver Metastasis. *Advanced Functional Materials*. 2021;31(42)doi:10.1002/adfm.202105089

22. Lu Z, Long Y, Li J, et al. Simultaneous inhibition of breast cancer and its liver and lung metastasis by blocking inflammatory feed-forward loops. *J Control Release*. Oct 10 2021;338:662-679. doi:10.1016/j.jconrel.2021.08.047
